# Hydrologic outputs generated over the Great Lakes with a calibrated version of the GEM-Hydro model

**DOI:** 10.1038/s41597-025-04409-x

**Published:** 2025-01-22

**Authors:** Étienne Gaborit, Juliane Mai, Daniel Princz, Hongren Shen, Vincent Vionnet, Bryan Tolson, Vincent Fortin

**Affiliations:** 1 Meteorological Research Division, Environment and Climate Change Canada, Dorval, QC Canada; 2 Department of Earth and Environmental Science, University of Waterloo, Waterloo, ON Canada; 3 National Hydrologic Services, Environment and Climate Change Canada, Saskatoon, SA Canada; 4 Department of Civil and Environmental Engineering, University of Waterloo, Waterloo, ON Canada

**Keywords:** Hydrology, Natural hazards

## Abstract

This dataset contains outputs from a calibrated version of the GEM-Hydro model developed at Environment and Climate Change Canada (ECCC) and is available on the Federated Research Data Repository. The dataset covers the basins of the Laurentian Great Lakes and the Ottawa River and extends over the period 2001–2018. The data consist of all variables (hourly fluxes and state variables) related to the water balance of GEM-Hydro’s land-surface scheme (including precipitation, surface and sub-surface runoff, drainage, evaporation, snow water equivalent, soil moisture…) and mean daily streamflow at 212 gauge locations. These outputs were simulated with a calibrated version of the GEM-Hydro model that was run in open-loop mode (no assimilation) and driven with atmospheric forcings coming from ECCC’s Canadian Surface Reanalysis version 2.1. GEM-Hydro achieves satisfactory simulations of various hydrologic variables when compared to reference datasets. This dataset can be used for example to drive any routing model, compute climatologies or statistics for different hydrologic variables and study their variability as a function of the local geo-morphology, etc.

## Background & Summary

The Laurentian Great Lakes basin (referred to as the Great Lakes) is a transboundary watershed between the United States (US) and Canada. It is the largest surface freshwater system on Earth and is home to 37 million people. Together with the Ottawa River basin, they represent a total drainage area of about 965 000 km^2^. For water resource management and in the context of climate change, it is important to understand the main processes governing the different components of the water balance in the region, as well as their spatial and temporal variability. Despite observations are being available for several components of the water balance, most of them only consist of *in-situ* measurements, which can be sparse both in space and time while being rarely co-located. Physically-based distributed hydrologic models can provide complementary information. Indeed, they produce consistent, seamless, and continuous (both in space and time) simulations of the different terms of the water balance equation, with a relatively detailed spatial and temporal resolution. These models can also be used for various applications, such as scenario testing or climate-change impact assessment.

The region of the Great Lakes has already been the target of a large number of studies that involved the implementation of physically-based distributed hydrologic models. For example, the studies of Lofgren (2004^1^), Pietroniro *et al*.^2^, Mao and Cherkauer^3^, Deacu *et al*.^4^, Warszawski *et al*.^5^, Mailhot *et al*.^6^, Mason *et al*.^7^, Do *et al*.^8^, and Mai *et al*.^9^ all involved the use of such models in this region. However, the simulations performed as part of these studies generally do not meet the requirements for the example uses mentioned in the abstract, like long-term hydrological assessments or driving/calibrating routing models. These former studies indeed involve at least one (but generally several) of the following limitations, they (i) only cover a relatively short time period (often 2 to 4 years), (ii) were performed at a low resolution (~40 km), (iii) did not cover the full Great Lakes area, (iv) involved model simulations forced with model precipitation (instead of observations or analyses), (v) involved a model that was not specifically calibrated for the region, and/or (vi) did not include a detailed hydrologic evaluation (sometimes streamflow performances were not computed, or computed based on monthly flows only). The other main limitation of most of these previous studies is that model outputs were not shared, or shared with a low spatial (only basin averages for example) or temporal resolution (only monthly averages for example), and for a limited number of hydrologic variables.

Some of the studies with the smaller number of previous limitations and for which hydrologic data were shared include the following: Livneh *et al*.^10^ implemented the VIC model over North America at a 6 km resolution, over the period 1950–2013, and driven by observed precipitation. As part of this study, many hydrologic variables were shared in a distributed manner and with a daily temporal resolution. However, the VIC model parameters were only calibrated for a few major watersheds (none directly located in the Great Lakes), by only targeting monthly flows, and transferred to other areas using an interpolation or nearest neighbor approach. Therefore, the VIC model parameters used in that study are most probably not optimal for the Great Lakes region. As part of the work described in Schellekens *et al*.^11^, 10 different Land Surface Schemes (LSSs) were implemented on a global scale, over the period 1979–2012, with a 40 km spatial and a 3-h temporal resolution. The models were forced with ERA-Interim reanalysis forcings. However, they were not calibrated and tend to display a high variability in snow-dominated regions such as the Great Lakes area, where snow is an important component of the hydrological cycle. The models were evaluated against reference datasets for several hydrologic variables except streamflow. The hydrologic data shared by Minallah *et al*.^12^ consist of outputs from the Noah-MP LSS (the LSS used in WRF-Hydro) and are available over the Great Lakes region, for the period 2016–2020, and with a 9 km and hourly resolutions. However, the model was not calibrated to maximize hydrologic performances in the region and the evaluation of the most important hydrologic variables like streamflow relied on monthly averages. Data from global land surface reanalysis such as ERA5-Land are also available over the region. ERA-5 Land (Muñoz-Sabater 2021^13^) has a global coverage with a 9 km resolution at hourly time step, over the period 1950-present, and relies on ERA-5 reanalysis forcings. The main limitation of this product is that it was not specifically calibrated to maximize hydrologic performances in the Great Lakes region. Note that Environment and Climate Change Canada (ECCC) is also preparing (at the time of writing) the distribution of the outputs of its Canadian Surface Reanalysis-Land (CaSR-Land) product, which is based on CaSR forcings (Gasset *et al*.^14^, covers North America with a 10 km and hourly resolutions over the period 1980–2017, but relies on a default (non-calibrated) version of GEM-Hydro.

Finally, the Great Lakes Runoff Intercomparison Project (GRIP-GL, see Mai *et al*.^15^ includes calibrated hydrologic models implemented over the full Great Lakes area (plus the Ottawa River basin) with a high spatial and temporal resolution, over the period 2001–2017, and forced with reanalysis forcings coming from CaSR (Gasset *et al*.^14^). As part of GRIP-GL, 4 hydrologic variables (streamflow, Surface Soil Moisture - SSM, EvapoTranspiration – ET, and Snow Water Equivalent - SWE) were evaluated with a daily temporal resolution and were shared for 12 participating models (13 for streamflow), which makes it a very valuable dataset. However, these 4 variables alone do not depict the full picture of each model’s water balance, and do not allow for driving a routing model, for example. To overcome the lack of a high-resolution and long-term well calibrated hydrological dataset over the Great Lakes’ region, GEM-Hydro (Gaborit *et al*.^16^, Vionnet *et al*.^17^) has been calibrated over this region. GEM-Hydro is a physically-based and distributed hydrologic model developed at ECCC, and was part of the GRIP-GL project. As part of GRIP-GL, GEM-Hydro showed competitive streamflow performances when compared to the other physically-based distributed models. It was moreover one of the best among all models regarding SWE, SSM and ET simulations. The dataset presented here corresponds to outputs of a new calibrated version of GEM-Hydro over the same region as the one used in GRIP-GL and over the period 2001–2018. This new version was developed to improve the performances of GEM-Hydro when compared to the version used in GRIP-GL, mainly regarding streamflow, SSM in the Lake Erie and Ottawa River basins, and to ET and SWE simulations in the Ottawa River basin (see results of GRIP-GL). At the same time, the dataset described here is more complete than the one shared as part of GRIP-GL for GEM-Hydro, since it includes all variables related to the water balance of the surface component of GEM-Hydro, with an hourly instead of a daily temporal resolution. Finally, the version of GEM-Hydro that was calibrated here allows to improve streamflow performances for the Great Lakes region while maintaining similar performances (compared to the default version of the model) for other hydrologic and surface variables, as demonstrated in this document with a detailed evaluation of some of the most crucial hydrologic and surface variables.

In the rest of this section, a brief overview of the GEM-Hydro model is given first, followed by explanations about the need to calibrate it and the associated challenges. A brief history of the GEM-Hydro calibration is then provided, before summarizing the methodology that was employed here to calibrate the version of GEM-Hydro whose outputs are shared in this dataset.

The GEM-Hydro model is used inside the National Surface and River Prediction System (NSRPS, see poster and extended abstract from Durnford *et al*. 2021, available at https://ams.confex.com/ams/101ANNUAL/meetingapp.cgi/Paper/383559). The NSRPS is used at ECCC to perform real-time surface and hydrologic analyses and forecasts, both deterministic and probabilistic, over Canada and the main US/Canada transboundary watersheds. The GEM-Hydro model is composed of two main components. The first one is GEM-Surf (Bernier *et al*.^18^), which is the surface component of the model. It can represent up to 5 different types of surface covers (or tiles) inside each grid cell, i.e. glaciers, land, water, frozen water, and urban areas. Over land, GEM-Hydro relies on the Soil, Vegetation and Snow (SVS) LSS (Alavi *et al*.^19^, Husain *et al*.^20^, Leonardini *et al*.^21^,^22^). SVS represents two types of snow-free covers, i.e., high and short vegetation, and bare ground. In winter, it represents two different snowpacks: snow over bare ground and short vegetation, and snow under high vegetation. SVS represents a single soil column made of several layers (generally 7, that go down to a depth of 3 m). In this study, GEM-Surf was implemented with a 10-km spatial resolution and a 10-min time-step. The second GEM-Hydro component is Watroute (Kouwen 2010^23^), which is the routing component of the model that is used to convey water in the network of lakes and rivers to simulate streamflow. Watroute is a gridded routing model implemented with a 1-km resolution in GEM-Hydro. It can represent lakes and reservoirs, as well as diversions. To represent regulated reservoirs, Watroute relies on the Dynamically Zoned Target Release (DZTR) reservoir model (Yassin *et al*.^24^, Gaborit *et al*.^25^). Watroute simulates baseflow (the contribution of the aquifers to the surface network) using a conceptual reservoir in each grid-cell that is called the Lower Zone Storage (LZS), and which is fed with drainage from GEM-Surf and estimates baseflow based on a power function. GEM-Surf also provides surface runoff and lateral flow to Watroute, which are provided as direct inputs to the network of lakes and rivers of Watroute. In GEM-Hydro, GEM-Surf and Watroute are one-way coupled, meaning that the routing scheme has no impact on the surface component. The bilinear interpolation method was used to interpolate GEM-Surf outputs at a 10-km spatial resolution and provide them as inputs to Watroute, which has a 1-km spatial resolution in GEM-Hydro. Please refer to Data records for more information on GEM-Surf outputs, and to Usage Notes for more information on the coupling between GEM-Surf outputs and any routing model. So far, the GEM-Hydro version used in NSRPS is the default version of the model, in the sense that none of its internal parameters were calibrated to maximize streamflow or other variables’ performances. Despite the default version of GEM-Hydro can have very satisfactory performances in some areas, it can also show very limited performances in others. Agricultural areas are a good example of regions where the default GEM-Hydro version has limited performances regarding streamflow simulations, because tile drains that are generally installed in such areas to drain any excess of water are not explicitly represented in the model. Like any physically-based model, it cannot accurately represent all of the physical processes unfolding in reality (Baroni *et al*.^26^, Budhathoki *et al*.^27^). Moreover, there are not enough field observations to accurately parameterize the model in all areas of a given domain (Hirpa *et al*.^28^). Finally, even physically-based models still rely on empirical or conceptual relationships in some parts of the model (Mai 2023^29^), for example to translate soil texture information into soil hydraulic conductivities. For all these reasons, any hydrologic model still needs calibration to achieve its best performances, as illustrated by many recent studies such as those of Budhatoki *et al*.^27^, Hirpa *et al*.^28^, Bajracharya *et al*.^30^, Mai 2023^29^, Mei *et al*.^31^, and Demirel *et al*.^32^, among many others.

GEM-Hydro is computationally expensive, which is generally the case for physically-based distributed hydrologic models, when implemented over large scales (Baroni *et al*.^26^. Therefore, the numerous simulations over long periods required for calibration can make it difficult (see for example Hirpa *et al*.^28^ to calibrate the model. In the case of GEM-Hydro, the model is not directly usable for calibration purposes when implemented over large regions such as the one considered here. The two main reasons for the high computational time of GEM-Hydro are that the surface component runs using successive 24-h integration cycles with a significant time spent in input/output processing, and that the Watroute routing scheme is gridded, implemented at a 1-km resolution, and not parallelized (all grid points are processed in a sequential manner from the most upstream points to the outlet).

Despite of this, GEM-Hydro parameters have already been calibrated in the past for research purposes, mainly as part of the different GRIP projects^9,15,16^. During these projects, computationally efficient alternatives to the components of GEM-Hydro were used to derive the relevant calibrated parameters. These alternatives have evolved in time. For example, GEM-Surf was replaced during calibration with a stand-alone SVS version and WATROUTE was replaced with a simple Unit Hydrograph technique in Gaborit *et al*.^16^. In GRIP-E (Mai *et al*.^9^, GEM-Surf was replaced with the “Modélisation Environnementale communautaire - Surface et Hydrologie” platform (MESH, see Pietroniro *et al*.^33^ including the SVS land-surface scheme and referred to as “MESH-SVS”, and WATROUTE was implemented with a coarse 10-km resolution. MESH-SVS is faster to run compared to GEM-Surf mainly because it runs over a given period using a single integration, i.e., not stopping and restarting each day like GEM-Surf does. Finally, in GRIP-GL (Mai *et al*.^15^, GEM-Hydro was fully replaced with MESH-SVS-Raven during calibration, which means that GEM-Surf was replaced with MESH-SVS and WATROUTE was replaced with the routing component of the Raven model (Craig *et al*.^34^). The calibrated parameters obtained with MESH-SVS-Raven were then transferred into the actual GEM-Hydro model. Raven routing is much faster than WATROUTE (see Mai *et al*.^15^) because Raven is a vector-based model. Therefore, the number of objects represented in Raven is generally much smaller (even with a high resolution) than the number of grid-cells in a 1-km WATROUTE setup. The Raven model is a flexible modelling platform offering various model choices for the water balance (most of which belong to the lumped and conceptual category of hydrologic models but that can be applied in a semi-distributed manner with a high resolution), as well as various modelling choices for the routing part. During GRIP-GL, only the routing component of Raven was used and coupled to MESH-SVS, in the same manner as Watroute or any other routing model would be coupled to MESH-SVS or GEM-Surf (see Usage Notes). We refer to Mai *et al*.^15^ for more information on the options and configuration of the routing component of Raven that was employed during GRIP-GL and used here as well.

**Fig. 1:**
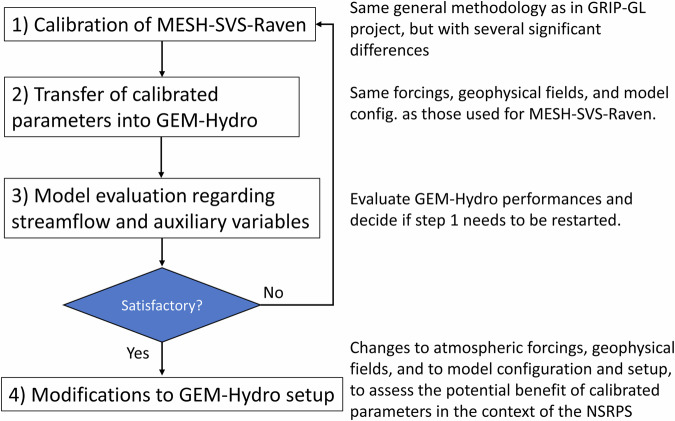
general workflow employed to calibrate some GEM-Hydro model parameters and use them in a setup that is close to the one used in the National Surface and River Prediction System (NSRPS). Note that the setup of step 4 is the one used to produce the outputs that are shared and described in this document. More details on the different steps of the methodology can be found in the Methods’ section.

The methodology that was employed here to calibrate GEM-Hydro is generally the same as the one used in the GRIP-GL project (see Mai *et al*.^15^) but it involves several significant differences compared to it. This new methodology is detailed in the Methods’ section, but a summary of the four main steps involved is presented further down and in Fig. 1.

The first step corresponds to calibrating a MESH-SVS-Raven model. The calibrated parameters obtained with MESH-SVS-Raven are then transferred into the actual GEM-Hydro model (step 2). For these first two steps, the atmospheric forcings, geophysical fields, and model configuration for both the GEM-Hydro and MESH-SVS-Raven models were the same as those used in the GRIP-GL project (Mai *et al*.^15^). This was done to be able to assess the benefit of the changes employed here for the GEM-Hydro calibration methodology when compared to the one employed during GRIP-GL (see next section). After the second step, a comprehensive evaluation of the calibrated GEM-Hydro version was performed including streamflow performances, but also auxiliary hydrologic variables and near-surface meteorological variables (see “Technical Validation”). This was done to ensure that the calibration exercise performed here by only maximizing streamflow performances did not degrade other hydrologic variables. If this was the case, changes were then brought to the calibration methodology and step 1 was restarted (see Fig. 1). After a total of six iterative calibrations performed during this study, the resulting GEM-Hydro performances were judged satisfactory, and step 4 was performed (Fig. 1). During step 4, the GEM-Hydro setup using the calibrated parameters was modified to use a setup that is close to the one employed in the operational version of NSRPS. This was done to more precisely assess the benefit that this calibration exercise could ultimately bring to NSRPS. Even if the NSRPS system differs in many aspects from the GEM-Hydro simulations performed in this study (different forcings, different spatial resolution…), and if NSRPS involves the assimilation of several variables whereas GEM-Hydro is run here in an open-loop mode, it is believed that the improvements that are brought in this study by the use of calibrated parameters will generally hold if used inside the NSRPS. Indeed, as shown in this study, the calibrated parameters consist of robust parameters that compensate for weaknesses of the GEM-Hydro model regarding some physical processes in the region, and do not aim at compensating for biases coming from forcings. Even if data assimilation can partially correct model errors in NSRPS, the longer-term forecasts will still be affected by GEM-Hydro model weaknesses, and (at least) these longer-term forecasts could therefore also be improved using a calibrated GEM-Hydro model.

The GEM-Hydro outputs shared in this dataset were obtained from step 4 of Fig. 1, which involves using a setup that is close to the one employed in NSRPS. More details can be found in next section. The dataset described here was published on the Federated Research Data Repository (FRDR; Gaborit 2024^35^). It is important to note that to produce these outputs, the calibrated version of the GEM-Hydro model was run in open-loop mode (no land-surface or streamflow assimilation performed) but was driven by atmospheric forcings coming from version 2.1 of CaSR (Gasset *et al*.^14^). Therefore, even if no assimilation was performed for any surface variable or streamflow during these calibrated GEM-Hydro runs, they were forced with atmospheric forcings that come from a reanalysis. This means that, for example, precipitation forcings correspond to precipitation analyses, which consist of short-term forecasted precipitation fields corrected by various sources of precipitation observations. The fact that no assimilation was performed in these GEM-Hydro runs has advantages. For example, the mass of water is conserved in these simulations (from total grid cell precipitation to total streamflow and evaporation leaving a watershed), whereas the general water balance can be significantly altered in the case of data assimilation, potentially leading to a decrease in model performance regarding streamflow simulations (see for example Garnaud *et al*.^36^). Another advantage is that without data assimilation, the physical link between the different simulated hydrologic variables is preserved, allowing for example to analyse their mutual relationships.

The outputs shared in this dataset consist of all variables (hourly fluxes and state variables) related to the SVS water balance (SVS consists of GEM-Hydro’s LSS) and includes mean daily streamflow time-series (observed and simulated values using WATROUTE), at gauge locations across the region of interest. The land surface variables consist of precipitation, surface runoff, sub-surface runoff (soil lateral flow), soil base drainage, ET, SWE, Soil Moisture (SM) content for 6 soil layers down to 2 m, and liquid water stored in the vegetation canopy. The outputs are provided over the Great Lakes and Ottawa River basins, and over the period from January 2001 to December 2018. The variables can be used, for example, to run and calibrate any routing model, compute climatology, various statistics, or trends for different hydrologic variables over the region of interest, to assess the variability of these variables as a function of the local geo-morphology, etc.

As part of the GRIP-GL project (Mai *et al*.^15^), GEM-Hydro was often the best model regarding SWE and SSM simulations, when compared to other conceptual and lumped models, or to other distributed and physically-based models widely used in North America, but the version presented here is even better regarding these variables. Therefore, it is argued that the hydrologic variables shared in this dataset can be useful to a broad variety of users in the hydrologic community.

## Methods

### Calibration: similarities with the GRIP-GL methodology

The general GEM-Hydro calibration methodology employed here is the same as in GRIP-GL (see Mai *et al*.^15^) and includes the main steps described hereafter. MESH-SVS-Raven is used during the calibration exercise in place of GEM-Hydro, to save computation time (see Background and Summary). The same geophysical fields and forcings as those used in the GRIP-GL project (Mai *et al*.^15^) were used with MESH-SVS-Raven during the calibration exercise performed here. The forcings come from the version 2.0 of the CaSR, which relies on RDRS v2.0 (see Gasset *et al*.^14^). For the MESH-SVS-Raven simulations used during calibration, a full year was used for model spinup (starting on January 1^st^ 2007), with model state variables (the most important ones because the longest to stabilize being snow mass and soil moisture content) initialized with fixed subjective values for all grid cells, equal to 300 mm for SWE and to 0.2 m^3^/m^3^ for SM content for all soil layers. Despite this can be seen as a crude initialization for a physically-based model, these values were chosen because they correspond to a significant amount of stored water at the start date for the region, and because it is generally faster for water to leave the model when it is in excess than to replenish where it is underestimated (especially for deep soil layers). It was moreover ensured that using different values (up to 0.3 m^3^/m^3^ for soil moisture content) did not significantly affect the value of the Objective Function (OF) computed over the calibration period. See further down for the initialization of the GEM-Hydro simulations.

**Fig. 2:**
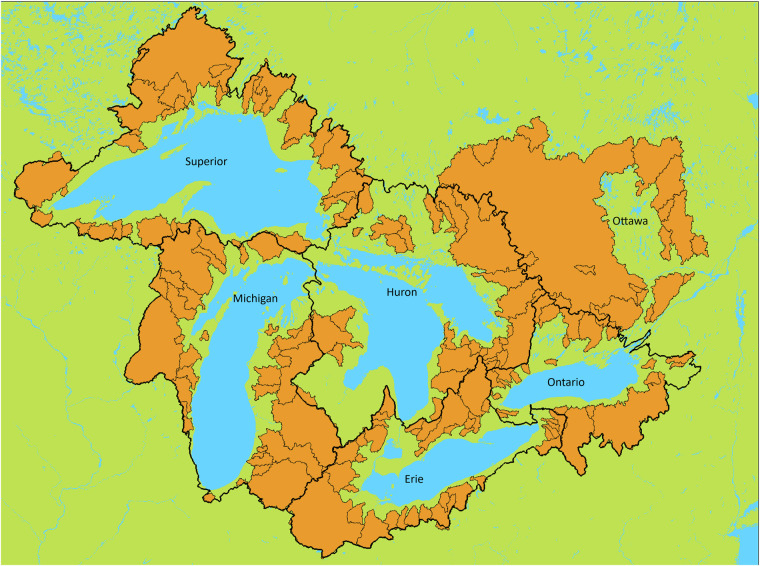
The delineation of the six main subdomains considered here (thick black lines show the 5 Great-Lakes watersheds, the rest belongs to the Ottawa River basin), along with the subbasins of the 212 flow gauges used in this study (orange).

A global calibration approach (Gaborit *et al*.^37^, Demirel *et al*.^32^) is used for each of the six main subdomains of the region of interest. There is a subdomain for each specific watershed of the five Great Lakes, plus the watershed of the Ottawa River, as shown on Fig. 2. This means that the performances at all flow gauges located inside a given subdomain are considered as a whole, for example using the median or the mean of the different stations’ performances. The only variable being considered to compute the OF for a given subdomain is the mean daily streamflow at flow gauges of this subdomain. This strategy of using a global calibration for each of the different subdomains is also referred to as “regional calibration” (Mai *et al*.^15^), or as “multi-basin calibration” (Demirel *et al*.^32^). Figure 2 presents the 6 main subdomains and the 212 basins considered in the region.

The Dynamically Dimensioned Search (DDS) calibration algorithm (Tolson and Shoemaker 2007^38^) is used with a maximum of 240 iterations to perform the calibration of MESH-SVS-Raven. Indeed, DDS is known for its fast convergence (Mai 2023^29^). Moreover, when looking at the evolution of the OF as a function of the number of iterations for any calibration experiment performed in this study, it was clear that the OF reached an asymptotical behaviour before the number of 240 iterations was reached (not shown). Note however that this number may be insufficient in other calibration contexts.

The calibration parameters mostly consist of multiplying coefficients that are used to multiply the actual model parameters (that generally vary in space) the same way to preserve their original spatial variability. The original actual model parameter values are computed by default by the model (SVS or Raven) based on their input geophysical fields. See Table 1 for information about the multiplying coefficients used as calibration parameters during this work. Some constraints were moreover applied inside the model to the adjusted parameter values, to make sure that the modified values would remain physically coherent. The final albedo values were for example capped to 1.0, and the final 50% root depth (see Table 1) was constrained to remain between a minimal value of 1 cm and a maximal value of 2 cm below the 95% root depth value.

**Table 1:** List of the calibration parameters used, along with a short definition.

		Init.	low	high	Parameter definition
MESH-SVS parameters	MLTM	1	0.8	1.1	snowmelt rate divider
	GRKMOD	1	1	2.5	horiz. hydraul. cond. multiplier (all soil layers, non-agric. areas)
	GRKMO_A	1	1	100	horiz. hydraul. cond. multiplier (layer 5, agric. areas)
	KASMOD	1	1	2.5	vert. hydraul. cond. multiplier (all soil layers, non-agric. Areas)
	KASMO_A	1	1	5.0	vert. hydraul. cond. multiplier (first 3 soil layers, agric. areas)
	EVMOD	1	0.8	1.5	evapo-transpiration resistance multiplier
	SUMOD	1	0.7	1.2	sublimation resistance multiplier
	RTMOD	1	1	1.5	95% and 100% root depth mutliplier
	DMOD	1	0.7	1.1	50% root depth multiplier
	AMOD	1	1	1.2	albedo multiplier
	URMO	1	1	1.3	urban area impervious fraction multiplier
Raven parameters	FLZCOEFF	2.40E-05	1.00E-07	1.00E-03	LZS multiplicative coeff.
	PWRC	2.8	2	4	LZS power coeff.
	R1NC	1	0.5	2	Mannings’ coeff. multiplier
	GASH	1	0.5	2	multiplier of the shape of the gamma UH
	GASC	1	0.5	2	multiplier of the scale of the gamma UH
	LACRWD	1	0.1	1	lake outlet width multiplier

Init.: Initial value; low,high: lower and upper limits of the interval allowed for a given parameter during calibration. horiz.: horizontal; hydraul.: hydraulic; cond.: conductivity; coeff.: coefficient; agric.: agricultural; LZS: Lower Zone Storage; UH: Unit Hydrograph. See text for the justification of interval limits used for some parameters.

### Calibration: differences regarding the GRIP-GL methodology

The differences between the methodology employed here to calibrate GEM-Hydro and the one used in GRIP-GL include the following changes. Bugs related to the reading of some geophysical fields in MESH-SVS were corrected (see Mai *et al*.^15^). Because of these bugs, the calibrated parameter values obtained with MESH-SVS-Raven during GRIP-GL were not optimal for GEM-Hydro, leading to a significant drop of performances between the calibrated MESH-SVS-Raven and GEM-Hydro simulations. Performances between the two systems are much closer now that the bugs have been fixed, as can be seen in the Technical validation section.

A total of 141 flow stations were used in GRIP-GL to calibrate the models, while 71 stations were kept for spatial validation. As many stations “as possible” were used here instead during calibration. This was done to ensure that almost all gauged catchments located in a given subdomain were taken into account in this subdomain’s OF. It was moreover not necessary to keep stations for the spatial validation of GEM-Hydro since its spatial robustness has already been demonstrated during GRIP-GL (Mai *et al*.^15^). Some stations were however still discarded here during calibration. Indeed, some of the basins considered here have a heavily regulated flow regime while this regulation was not explicitly represented in the Raven model employed here. For some regulated catchments, this resulted in poor model performances (see Table 2) that would have impacted the value of the OF (since it is based on the average of all local performances in each subdomain). Ultimately, including these problematic locations in the OF’s computation could have misguided the evolution of the calibration algorithm. In some cases, the regulation however only had a relatively limited impact on MESH-SVS-Raven flow simulations. This is the case for example for the station 02KF005 (Ottawa River at Brittania): despite the basin includes about 12 major dams, the regulation still has a limited impact on the total flow of the watershed (especially in spring), due to its large size (90 900 km^2^). The list of stations that were excluded from the calibration exercise performed here can be found in Table 2. All stations were however considered when evaluating the default and calibrated versions of the GEM-Hydro model (see Technical validation), because many dams are explicitly represented with the DZTR model in the Watroute model, and because GEM-Hydro was not used for calibration purposes. The complete list of the 212 flow stations considered in this study, along with their main basin characteristics, can be found in Mai *et al*.^15^.

**Table 2:** List of stations not considered during calibration, along with their (revised) Kling-Gupta Efficiency (KGE, see Kling *et al*.^39^) performances over the period 2001–2010 for the default version of MESH-SVS-Raven, and the default and calibrated versions of the GEM-Hydro model.

Subdomain	Station ID	Watershed size (km^2^)	Default MESH-SVS-Raven	Default GEM-Hydro	Calibrated GEM-Hydro
Huron	02EB011	4790	−1.58	0.35	0.52
Huron	02DD010	13900	0.47	0.63	0.63
Huron	02DB005	3150	0.11	0.3	0.36
Huron	04136000	2870	−0.44	−1.41	−0.59
Huron	04137500	4500	−0.77	−1.67	−0.81
Ont	02HF002	1280	−0.02	0	−0.2
Ott	02LE025	883	−0.11	0.02	0.08
Sup	02AD012	24700	0.42	0.75	0.7
Sup	02BD002	5310	−0.1	−0.1	−0.35
Sup	02BD007	1950	0.13	0.21	0.01
Sup	02BE002	2880	−0.31	−0.24	−0.48
Sup	04044724	210	0.32	0.05	−0.07
Median of the five underlined stations			0.11	0.35	0.52
Median of other stations			−0.10	−0.10	−0.35

Underlined stations are those for which the regulation is explicitly represented in GEM-Hydro using the DZTR model (Yassin *et al*.^24^). Note that in the MESH-SVS-Raven setup used here, the DZTR model was not used for any stations.

For each subdomain, the OF considered during calibration consists of the mean of the normalized (revised) Kling-Gupta Efficiency (KGE) criteria (see Kling *et al*.^39^) across the flow stations of this subdomain. Based on previous calibration experiments performed, it was preferred to use the mean than the median. When using the median, some station performances are always not reflected in the OF, resulting into neglecting their performances. All stations’ performances are however considered in the OF when using the mean. A normalized version of the (revised) KGE criteria was therefore required. It corresponds to the revised KGE, but rescaled such that it falls between 0 and 1. This is done to prevent large negative KGE values obtained for some stations from strongly affecting the mean, which would put too much emphasis on the worst stations. To normalize the KGE, Eq. 1 below was used:

1 $${KGEn}=\frac{1}{2-{KGE}}$$

with

${KGEn}:$ normalized KGE criteria (values between 0 and 1)

${KGE}:$ revised KGE criteria (Kling *et al*.^39^, values between −∞ and 1).

The KGE criteria was employed for the OF because it allows to take several aspects of the streamflow simulations into account through its three components (i.e. correlation, bias and variability, see Kling *et al*.^39^), without putting too much emphasis on some aspects of the hydrographs like peak flows. Indeed, the NSRPS system used at ECCC and relying on the GEM-Hydro model aims at fulfilling various needs and therefore must perform as well as possible both for low and high flow periods.

The calibration was performed over the period 2008–2017 included (2007 used as spin-up), and the validation period spans over 2001–2007 included (2000 used as spin-up). Using a more recent calibration period was preferred given that the calibrated parameters are to be used ultimately in the NSRPS real-time forecasting system and because the land use/land cover databases used here correspond to more recent periods as well (see Changes to the surface component further down).

Some calibration parameters used in GRIP-GL were discarded here because the auxiliary hydrologic variables were too sensitive to their variations (see Technical validation”). These parameters are: (i) the multiplying coefficients related to the three SM content thresholds (wilting point, field capacity and saturation), (ii) the slope of the soil water retention curve, (iii) the soil water suction at saturation, (iv) the vegetation leaf-area index, and (v) the vegetation roughness (see Mai *et al*.^15^). Note that the identification of these problematic parameters was progressively done during the 6 total iterations of the calibration exercise (see further down and Background and summary). The final lower and upper limits for some parameter intervals mentioned in Table 1 are also different than the values used in GRIP-GL because they were also progressively refined during these 6 iterations. The refinement of parameter intervals allowed during calibration was performed either to prevent unrealistic resulting parameter values in the GEM-Hydro model, to limit the degradation sometimes noticed for some flow stations or some auxiliary variables (see Technical validation), or simply to discard some parameter ranges that were never chosen as the best values by the calibration algorithm, with the objective to prevent the calibration algorithm to explore useless regions for some parameter values. The latter case for example explains why the lower bound for some parameters of Table 1 are sometimes equal to the initial value of 1.0.

A new approach to represent the effect of tile drains was employed during calibration: a specific calibration parameter was used to increase the horizontal hydraulic conductivity of the 5^th^ SVS soil layer in agricultural areas. This 5^th^ layer is located between the depths of 40 and 100 cm, where agricultural tile drains are generally located. This approach to represent the effect of tile drains in large-scale hydrologic models was suggested by De Schepper *et al*.^40^. A unique soil column is however used in SVS regardless of the vegetation cover type. A unique multiplying coefficient therefore needs to be used to increase hydraulic conductivity. This is done by computing a weighted average of two multiplying coefficients, one specific to agricultural covers and the other one for other covers (see Table 1), as mentioned in Eq. 2 below:

2 $${GRKM}\left(I,K\right)={GRKMOD}\left(I\right)\ast \left(1.0-{FRA}{C}_{A\left(I\right)}\right)+{GRKMO}{\rm{\_}}A(I)\ast {FRAC}{\rm{\_}}A(I)$$

where:

*GRKM*(*I*, *K*): final multiplying coefficient used to adjust the soil horizontal hydraulic conductivity of soil layer *K*, in grid-cell *I*. Note that Eq. 2 above is only used for *K* = 5 (5^th^ soil layer). For the other layers, *GRKM*(*I*, *K*) = *GRKMOD*(*I*)

*GRKMOD*(*I*): multiplying coefficient used to adjust soil horizontal hydraulic conductivity for vegetation covers other than the agricultural type, in grid-cell *I*.

*GRKMO_A*(*I*): multiplying coefficient used to adjust soil horizontal hydraulic conductivity for agricultural cover, in grid-cell *I*.

*FRAC_A*(*I*): fraction of the land tile occupied by agricultural cover in current grid cell *I*.

A new approach was also used to represent the effect of ploughing: a specific calibration parameter was used to increase the vertical hydraulic conductivity of the first three SVS soil layers in agricultural areas (i.e., for depths between 0 and 20 cm, which are generally impacted by ploughing). For the reasons explained above, this specific coefficient related to agricultural cover however needs to be merged with the coefficient related to other covers, following Eq. 3 below.

3 $${KASM}\,(I,K)\,={KASMOD}\,(I)\,\ast \,(1.0-{FRA}{C}_{A(I)})\,+{KASM}{\rm{\_}}A(I)\ast {FRAC}{\rm{\_}}A(I)$$

where:

*KASM*(*I*, *K*): final multiplying coefficient used to adjust the soil vertical hydraulic conductivity of soil layer *K*, in grid-cell *I*. Note that Eq. 3) above is only used for *K* = 1 to 3 (first 3 soil layers). For the other layers, *KASM*(*I*, *K*) = *KASMOD*(*I*)

*KASMOD*(*I*): multiplying coefficient used to adjust the soil vertical hydraulic conductivity for vegetation covers other than the agricultural type, in grid-cell *I*.

*KASM*(*I*, *K*): multiplying coefficient used to adjust soil vertical hydraulic conductivity for agricultural vegetation cover, in grid-cell *I*.

*FRAC_A*(*I*): fraction of the land tile occupied by agricultural cover in current grid cell *I*.

**Fig. 3:**
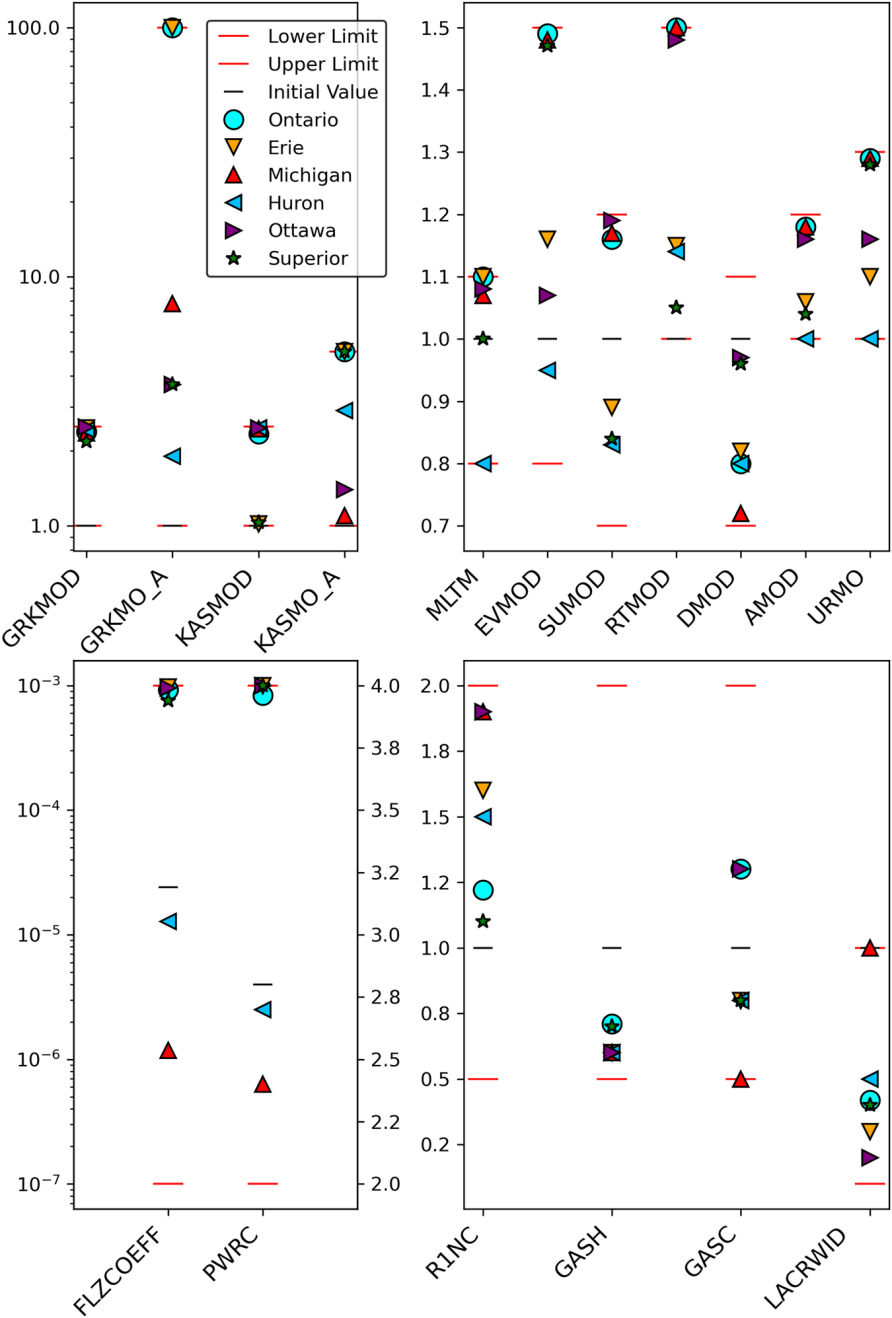
final calibrated parameter values for each subdomain, along with the initial values and the lower and upper interval limits used for each parameter. The top graphs show the SVS parameters, while the bottom graphs show the calibration parameters for the Raven routing model. See Table 1 for a description of the parameters shown here. Note that for the lower left graph, two different y axes are used for each parameter.

The final values of the calibrated parameters mentioned in Table 1 are shown for the 6 different subdomains on Fig. 3. It can be seen on this figure that some calibrated parameters exhibit some spatial consistency between the domains regarding their evolution, because they were generally all increased (or decreased) compared to their default value, which is the case for example for GRKMOD, KASMOD, RTMOD, DMOD, R1NC, GASH, and LACRWID. See Table 1 for a definition of the different calibration parameters. On the opposite, some parameters evolved differently compared to their default value depending on the subdomain, which is the case for MLTM, EVMOD, SUMOD, FLZCOEFF, PWRC, and GASC. In the case of the two parameters related to the LZS (FLZCOEFF and PWRC), their final values for the Lake Huron and Lake Michigan subdomains are very different than those chosen for the other subdomains, which moreover display a very strong coherence between them. This could be the indication of hydrogeologic processes that are specific to the Lake Huron and Lake Michigan subdomains, for example related to the existence of deep (confined) aquifers in these areas. The simple LZS conceptual model used in Watroute to simulate baseflow may therefore not catch the actual processes occurring for these subdomains, which would probably require a more complex hydrogeologic model. The fact that the GRKMO_A parameter displays a large spread for calibrated values may be because some subdomains contain almost no agricultural areas (like the Lake Huron, Lake Superior and Ottawa River subdomains), resulting in these domains’ OF to be insensitive to this parameter during calibration. This is also probably the case for the URMO parameter related to the fraction of impervious surfaces in urban areas: only the Lake Ontario and Lake Erie subdomains contain basins that are significantly impacted by urban areas. Finally, some parameters reached the upper or lower limit of their allowed interval (see for example GRKMOD, KASMOD, RTMOD, EVMOD, FLZCOEFF or PWRC), but constraining them was done on purpose as explained previously.

### Transfer of calibrated parameters into GEM-Hydro

Once the calibration was performed with MESH-SVS-Raven, the calibration parameters were transferred into the actual GEM-Hydro model (see step 2 of Fig. 1), with no change to the configuration, setup, forcings, etc., such that differences between the calibrated MESH-SVS-Raven and GEM-Hydro models would mainly come from the differences related to the change of the routing model (i.e., from Raven in MESH-SVS-Raven to Watroute in GEM-Hydro). This was done to ensure that the two different modelling platforms were leading to similar results, and that the calibrated parameters obtained with MESH-SVS-Raven were appropriate for use in GEM-Hydro. See the Technical validation section for the differences between MESH-SVS-Raven and GEM-Hydro performances.

It is important to emphasize that Raven and Watroute are two different routing schemes. Therefore, even if some parameters calibrated using Raven could be directly transferred into Watroute (such as the two FLZCOEFF and PWRC parameters related to the groundwater discharge computation, see Table 1), the others could not be transferred because some processes were not represented the same way in the two routing schemes. Raven for example relies on a convolution Hydrograph (parameters GASC and GASH, see Table 1) to simulate the transport of a grid cell’s runoff into the grid-cell’s main stream, while Watroute assumes that this transport is “instantaneous”. The parameter “LACRWD” (Table 1) could not be transferred into Watroute since Raven and Watroute do not (yet) rely on the same equation to simulate outflow from natural lakes. Therefore, the other Watroute parameters remained unchanged compared to the default version of the model. An attempt was nevertheless made to further tune the Watroute Manning roughness coefficients by manually adjusting these values for each of the 6 subdomains with the calibrated GEM-Hydro version. Since no significant performance gain could however be obtained this way, it was preferred to use the default Watroute values for these parameters.

The GEM-Hydro simulations started on January 1^st^, 2000, and were initialized with GEM-Hydro outputs corresponding to January 1^st^ of 2014. These GEM-Hydro outputs are the result of a former open-loop run with the default model version and over multiple years (from 2000 to 2014). The year 2000 was considered a spinup period for the model runs and was not used for evaluation (the dataset described here does not include this spinup year). Despite the year 2014 may not be the best choice to initialize GEM-Hydro in 2000 because of differences in terms of climatic conditions between the two periods, it is still a preferable choice than using fixed values for all grid-cells as was done with MESH-SVS-Raven. The GEM-Hydro initial conditions indeed vary in space for each grid-cell and therefore reflect the heterogeneities of the region in terms of soil texture, vegetation, etc. The GEM-Hydro simulations correspond to continuous simulations over the full period of the study (2000–2018 included), and it generally only takes about two years for the soil moisture of the last soil layer considered here (down to 2 m) to stabilize, based on experience with the model.

### Model evaluation regarding streamflow and auxiliary variables

At this stage, a comprehensive evaluation of the GEM-Hydro auxiliary hydrologic variables and near-surface variables (SWE, SSM, ET, 2-m temperature and dew point, 10-m wind speed) was performed (see Fig. 1 and Technical validation) in order to make sure that the calibration exercise, only focused on maximizing streamflow performances, did not degrade hydrologic or near-surface variables when compared to the performances of the default GEM-Hydro version. If this was the case, then the first step of Fig. 1 was restarted after changes were brought to the calibration methodology. Most of these changes were related to the parameters used during calibration and/or to their intervals, as explained in “Calibration: Differences with regard to the GRIP-GL calibration methodology”. A total of six iterations of the cycle mentioned on Fig. 1 was needed in this study before performances for the auxiliary variables were judged satisfactory. Making sure that hydrologic variables other than streamflow, as well as surface variables and fluxes, are not degraded compared to the default version of the model is important for two main reasons. The first reason is related to the fact that the goal of this calibration exercise is to use a calibrated GEM-Hydro version in the NSRPS, where various data are being assimilated such as snow cover, SSM, and surface temperatures. Simulation performances therefore need to at least be maintained for these variables, or the assimilation process may be significantly altered. The second reason has to do with the goal of the SVS land-surface scheme, which is to be used directly in the atmospheric systems used at ECCC, with the long-term vision of using the same systems both for Numerical Weather Prediction (NWP) and hydrologic forecasts. As such, calibrating SVS to optimize streamflow performances should not lead to degrading the screen-level temperatures and fluxes or this could have a negative impact on weather forecasts.

### Modifications to the GEM-Hydro setup

Significant changes were then brought to the GEM-Hydro setup, but still using the same calibrated parameters (see step 4 of Fig. 1), to assess the potential benefit that the calibrated parameters could bring when employed in the NSRPS system. This modified setup was the one used to produce the GEM-Hydro outputs that are being shared in this dataset. More details about this “final” GEM-Hydro setup are provided below. A comprehensive evaluation of the GEM-Hydro performances obtained after this step was also performed to make sure that no significant degradation was noticed for streamflow, auxiliary hydrologic and near-surface variables (see Technical validation), when compared to the GEM-Hydro setup used during step 2 (see Fig. 1). Table 3 below summarizes the main modifications brought to the GEM-Hydro setup.

**Table 3:** Main differences between the GEM-Hydro setup used during step 2 of Fig. 1 (the “GRIP-GL” setup) and the one used during step 4 (the “NSRPS” setup).

	Forcings	Calibrated parameters	Geophysical fields	Modelling option	Model version
GRIP-GL setup (step 2)	CaSR v2.0	Fixed for each subdomain	GRIP-GL	Precip. PPM: 0-degree threshold	GEM-Surf 6.1.2
NSRPS setup (step 4)	CaSR v2.1	Smoothed at subdomain boundaries	NSRPS	Precip. PPM: Harder and Pomeroy (2013^41^)	GEM-Surf 6.2.0

^1^see Mai *et al*.^15^ for more information about the geophysical fields used during the GRIP-GL project. Precip. PPM: precipitation phase partitioning method. See text for more details about the NSRPS setup. See Fig. 1 for the different steps of the methodology employed here.

#### Changes to the surface component

GEM-Surf version 6.2.0 was used. The different GEM-Surf options of this “final” GEM-Hydro setup are not described here in detail. We refer to the readme file of the dataset (Gaborit 2024^35^) for the list of the options used. One important change, however, is that this final setup is using the precipitation Phase Partitioning Method (PPM) of Harder and Pomeroy (2013^41^) whereas the previous setup used a 0°C. air temperature threshold to split total precipitation into rainfall and snowfall. Using a humidity-based PPM such as Harder and Pomeroy (2013^41^) strongly improved the ability of GEM-Hydro to predict the precipitation phase (Vionnet *et al*.^42^). Atmospheric forcings come from version 2.1 of the CaSR (Gasset *et al*.^14^). Regarding the geophysical fields, the final GEM-Surf setup relies on the following data sources, which for some of them differ from the sources used during GRIP-GL (see Mai *et al*.^15^).

- The Global Multi-resolution Terrain Elevation Data version 2010 (GMTED 2010, see USGS, 2010^43^) was used for surface topography (elevation, slope) and Watroute elevation data (different from the one used in GRIP-GL).
- The Climate-Change Initiative – Land Cover dataset version 2015 (CCI-LC 2015, available for download at https://maps.elie.ucl.ac.be/CCI/viewer/download.php) was used for land use/land cover in GEM-Surf and Watroute (different from the one used in GRIP-GL).
- The Global Soil Dataset for Earth system modelling (GSDE, see Shangguan *et al*.^44^) was used for soil texture.
- The National Hydrographic Network (NHN, available at https://open.canada.ca/data/en/dataset/a4b190fe-e090-4e6d-881e-b87956c07977) and the National Hydrographic Dataset (NHD, available at https://www.usgs.gov/core-science-systems/ngp/national-hydrography/national-hydrography-dataset) were used for drainage density.
- The HydroSHEDS 30 arcsec. (~1 km) resolution dataset (Lehner *et al*.^45^) was used for WATROUTE flow direction grids (dataset available at https://www.hydrosheds.org/hydrosheds-core-downloads).

In opposition to the actual NSRPS configuration, and similarly to the GRIP-GL configuration of GEM-Hydro, subgrid-scale lakes were however still deactivated in the final configuration used here. This means that when a grid cell contains less than 100% water, the water tile inside that grid-cell is not considered by the model and is replaced by the other surface tiles of the grid cell while preserving their relative importance. Note that in the region of this study, only two types of surface tiles were present: Land surface and water (including ice-free and frozen water), because no glaciers are present and because the explicit representation of urban areas using the Town-Energy-Balance (TEB) model was not activated, such that urban areas are included in the land-surface tile, in this setup. Therefore, neglecting the water tile for pixels with less than 100% of water resulted here in considering that the pixel was covered with 100% land.

Grid-cells containing 100% of water were however not modified as no other tile could replace it. This “filtering” of water surfaces was also not done for grid cells located directly around pixels containing 100% water. The variable “WT” shared in this dataset (see Data records) indicates the fraction of land in each grid-cell of the region, the rest corresponding to the fraction of water in the grid-cell. This filtering is needed because with the model currently used in GEM-Hydro to represent water surfaces, an external source must be used for water temperature and ice fraction. Generally, an ECCC internal analysis is used for that, but it is only available from 2010 or so and not over the whole period of this study. Therefore, ERA-Interim was used to provide this information over the full period used here (2000–2018), for consistency throughout the whole period. Based on tests performed with this source, it is however not judged reliable in terms of the resulting evaporation simulated over water with GEM-Hydro. It was therefore preferred to neglect the water portion of the grid-cells where possible, leading to filtering out the subgrid-scale lakes from the GEM-Hydro setups used during this study. This is also why only the hydrologic variables over land are provided in this dataset, and not over water surfaces (see Data records). Note that neglecting the subgrid-scale lakes in GEM-Hydro however has a limited impact on the resulting streamflow simulations of the region, based on tests previously performed (not shown here) and because ET over land is not so different than ET over water, inside the same grid-cell and when using ECCC internal analysis for water temperature and ice fraction.

**Fig. 4:**
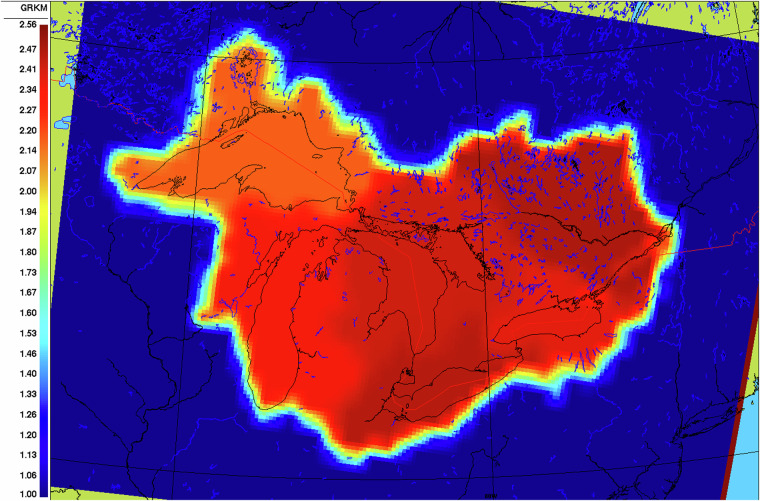
example smoothed 2-D field of the GRKMOD calibrated parameter values that were provided as input to GEM-Hydro when using a single model setup over the full Great-Lakes and Ottawa River domains. Outside of these domains, the default (uncalibrated) value of 1.0 is applied to this parameter. See Table 1 for the definition of the GRKMOD parameter.

The GEM-Surf calibrated simulations were performed during step 4 (Fig. 1) using a single model setup covering the whole geographic region of interest (i.e., all the 6 subdomains were included in this single setup, while separate setups were used for each subdomain during step 2 of Fig. 1). To do so, the calibration parameters (i.e. the multiplying coefficients of Table 1) were provided as 2-D input fields to GEM-Surf (similarly to other static geophysical fields). To avoid abrupt parameter changes at subdomain boundaries, the 2-D parameter fields were however smoothed. Not doing so could ultimately create abrupt changes in some surface fluxes or variables at these boundaries, which is not desirable in the (future) context of coupling this calibrated version with an atmospheric model. To smooth the calibrated parameter values, the 2-D fields of the fixed parameters values for each subdomain were combined and then aggregated by a factor of 3 (i.e., decreasing the resolution by a factor of 3), before being bilinearly interpolated back on the original grid resolution. Figure 4 shows an example smoothed 2-D field for the GRKMOD calibrated parameter.

#### Changes to Watroute

The ECCC version 3.4 of Watroute was used. Similarly to the GEM-Surf component, as part of the “final” GEM-Hydro open-loop run, Watroute was run using a single setup over the full Great Lakes and Ottawa River region. To do so, the two parameters that were calibrated with Raven and transferred into Watroute were also provided to Watroute as 2-D static fields. Note that in this case, no smoothing of the parameter values at subdomain boundaries was however required, because the surface and routing components of GEM-Hydro are one-way coupled, such that the routing component cannot have any impact on the surface component (and therefore on the atmospheric model).

### Methods for processing the GEM-Hydro outputs

#### Surface fluxes (precipitation, ET, surface runoff, lateral flow and drainage, in mm/h)

GEM-Hydro runs over long periods by performing 24-h cycles of continuous integrations, between 12:00 UTC and 12:00 UTC the day after. For each of these 24-h cycles, the raw output fluxes consist of accumulated values in mm since the start of the 24-h integration. These raw outputs were then processed to compute the “decumulated” fluxes, such that each flux provided in this dataset consists of the quantity of water (in units of kg/m^2^, or mm) over the hour preceding the date mentioned. All dates of the GEM-Surf variables shared in this dataset correspond to time in the Universal Time Coordinated (UTC) format. For all the GEM-Surf outputs, the standard files (a binary format used internally at ECCC) were all converted into the netcdf format.

#### Snow Water Equivalent (SWE, in mm)

GEM-Hydro does not directly output the mean SWE over the land part of a grid-cell. GEM-Hydro (SVS) does simulate two different snowpacks over the land area of a grid cell: one under high vegetation, and one over bare ground/ short vegetation (Leonardini *et al*.^22^). The mean land SWE in a grid cell was computed from the raw outputs using Eq. 4 below:

4 $${\rm{SWE}}=({\rm{SNDP}}\ast {\rm{SNDN}}\ast 0.01+{\rm{WSN}})\ast (1.-{\rm{VEGH}})+({\rm{SVDP}}\ast {\rm{SVDN}}\ast 0.01+{\rm{WSV}})\ast {\rm{VEGH}}$$

where:

SNDP, SVDP: snowpack depth (cm) respectively over bare ground + short vegetation, and under high vegetation.

SNDP, SVDP: snowpack density (kg/m^3^) respectively over bare ground + short vegetation, and under high vegetation.

WSN, WSV: liquid water stored in the snowpack (mm), respectively over bare ground + short vegetation, and under high vegetation.

VEGH: fraction of the land area of the grid cell that is covered with high vegetation.

#### Water stored in/on vegetation (WVEG, in mm)

In SVS, the quantity of water stored in/on vegetation is valid for the fraction of the land surface covered by snow-free vegetation (high vegetation and snow-free low vegetation). The raw WVEG SVS output was therefore modified according to Eq. 5 below, such that it corresponds to a height of water (in mm) valid over the whole land surface area, in order to be used directly in Eqs. 6 and 7 to compute the SVS water balance over the land-surface area of a grid-cell (see Data Records).

5 $${WVEG}={{WVEG}}_{{raw}}\ast (1-{PSGL})$$

where:

*WVEG*: final WVEG variable shared in this dataset, and valid over the whole land surface area of a grid-cell (in mm)

*WVEGraw*: raw SVS output corresponding to the quantity of water (in mm) stored in the vegetation fraction of a grid-cell’s land tile that is above the snowpack.

*PSGL*: fraction of the land surface that is covered with snow over bare ground and snow over low vegetation.

#### Soil moisture for each soil layer (WSL 1–6, m^3^/m^3^)

No manipulation of the raw soil moisture outputs was performed but information is provided here on how to convert the values provided in this dataset. Soil moisture has units of m^3^/m^3^: it represents the fraction of a given soil layer that is filled with liquid water (the version of SVS used here does not represent freeze/thaw processes). To convert soil moisture from m^3^/m^3^ into soil moisture with units of mm (needed to compute the SVS water balance for example, see Eqs. 6 and 7), one needs to multiply the soil moisture content (m^3^/m^3^) for a given soil layer by the thickness of this soil layer in mm. Then, one could sum up the soil moisture over the six soil layers to obtain the total amount of water stored in the SVS soil column, in units of mm. The depth of the SVS soil layers’ interfaces and their associated thickness are mentioned in Table 4. The total soil thickness in this GEM-Hydro configuration is equal to 2000 mm (2 m).

**Table 4:** Depths of the SVS soil layer interfaces (in cm) and their thicknesses (in mm).

Soil Layer Number:	Depth (from surface) of upper interface (cm)	Depth (from surface) of bottom interface (cm)	Thickness (mm)
1	0	5	50
2	5	10	50
3	10	20	100
4	20	40	200
5	40	100	600
6	100	200	1000

For all the GEM-Surf outputs (2D gridded fields) provided in this dataset (except the WT variable), the fields were then filtered to remove the values located outside of the region of interest, i.e. outside of each of the five Great Lakes watersheds, and outside of the Ottawa River watershed. This was done because the calibrated parameter values only apply inside of these watersheds. Therefore, outside of them, the GEM-Hydro outputs correspond to the default version of the model. It was preferred not to mix outputs corresponding to the default version of the model with outputs corresponding to the calibrated version of the model in the same files included in this dataset. The values filtered out were replaced by “NaN” values. However, the WT variable is not simulated by the model and corresponds to a static field that represents the fraction of the grid-cell occupied by land in the setup used here. Therefore, it was not filtered out outside of the region of interest like the other surface variables. It is reminded that subgrid-scale lakes were deactivated here (see Modifications to the GEM-Hydro setup) such that most of the grid cells outside of the big lakes have a WT value (land fraction) equal to 1.0 here (100% land).

#### Streamflow (m^3^/s)

The Watroute component of GEM-Hydro produces streamflow at a 1-km resolution and with an hourly time-step. In this dataset, we however only provided simulated and observed streamflow time-series for the grid cells containing a flow gauge. The hourly flows were moreover converted into mean daily flows because a daily time-step was used to evaluate model performances regarding streamflow (see further down for justification). The daily flows shared in this dataset for a given date are valid between 00:00 and 00:00 the day after in local time, i.e. from midnight to midnight, with time always corresponding to the Eastern Daylight Time (EDT, corresponding to UTC minus 4 hours). More information on the 212 flow gauges for which mean daily flows are reported in this dataset can be found in Mai *et al*.^15^, on the USGS website (https://waterdata.usgs.gov/nwis/dv/?referred_module=sw), or on the Water Survey of Canada (WSC) website (https://wateroffice.ec.gc.ca/search/historical_e.html). The US streamflow observations included in this dataset come from this USGS website. The Canadian daily streamflow observations were obtained from the HYDAT database (https://www.canada.ca/en/environment-climate-change/services/water-overview/quantity/monitoring/survey/data-products-services/national-archive-hydat.html).

Despite GEM-Hydro produces hourly streamflow simulations, it was preferred to perform the evaluation at a daily temporal resolution than at an hourly resolution. It is indeed challenging to gather flow observations at their original (sub-hourly) temporal resolution from the US and Canadian websites mentioned above (especially over long and not recent periods). A comparison between a daily and an hourly evaluation of streamflow performances was however conducted over the year 2011 for three small catchments of the studied domain (not shown here). The conclusion is that there is no significant difference between scores computed either at a daily or hourly temporal resolution and for any of the GEM-Hydro versions (i.e., default or calibrated), supporting the choice of the daily time-step employed here for the streamflow verification. Finally, it is emphasized that performing the streamflow evaluation at a daily temporal resolution is already an improvement compared to many other studies that employed physically-based hydrologic models over the region, as explained in the Background and Summary section and for which streamflow performances are often not evaluated or evaluated with a monthly time-step only.

## Data Records

The dataset is available at the Federated Research Data Repository (see Gaborit 2024^35^), with this section being the primary source of information on the availability and content of the data being described. This dataset consists of outputs from a calibrated version of the GEM-Hydro hydrologic model, which is a physically-based and fully distributed model. The dataset covers the period from 2001-01-01 to 2018-12-31. The outputs are available over the Canadian/US watersheds of the Great Lakes (Lake Superior, Lake Michigan, Lake Huron and Georgian Bay watershed, Lake Erie and Lake Saint-Clair watershed, Lake Ontario watershed), and over the Ottawa River watershed (Canada, provinces of Ontario/Québec). These outputs consist of all variables (hourly fluxes and state variables) related to the water balance (see Eqs. 6 and 7 below) of the land-surface tile (simulated with the SVS LSS) of the surface component of GEM-Hydro (named GEM-Surf), and of the mean daily streamflow time-series (observed and simulated with the Watroute routing component of GEM-Hydro), at the 212 gauge locations of the region of interest.

The equations of GEM-Hydro land-surface (SVS) water balance can be written as follows for any grid-cell and over any temporal period:

6 $$\varDelta S={PR}-({ACWF}+{TRAF}+{ALAT}+O1)$$

with:

$\varDelta S$: Change in storage (final storage – initial storage) between the final and initial dates of the period being considered, in mm (or kg/m^2^). See equation 7 for how to compute the SVS water storage.

${PR},{ACWF},{TRAF},{ALAT},O1$: Accumulated values of the different SVS water fluxes (see Table 5) over the period being considered, in mm or kg/m^2^.

**Table 5:** list of files shared in this dataset and description of their content.

Filename (Hydrologic variable)	Hydrologic Variable	Units	Definition
GEM-Hydro_calibrated_PR.nc	PR	mm/h	Hourly total precipitation over the hour preceding the date mentioned
GEM-Hydro_calibrated_ACWF.nc	ACWF	mm/h	Hourly evapo-transpiration over land surface over the hour preceding the date mentioned
GEM-Hydro_calibrated_TRAF.nc	TRAF	mm/h	Hourly surface runoff over land surface over the hour preceding the date mentioned
GEM-Hydro_calibrated_ALAT.nc	ALAT	mm/h	Hourly total lateral flow from land surface soil column (from all active soil layers) over the hour preceding the date mentioned
GEM-Hydro_calibrated_O1.nc	O1	mm/h	Hourly drainage from (vertical water flux leaving the) land surface last active soil layer over the hour preceding the date mentioned
GEM-Hydro_calibrated_WVEG.nc	WVEG	kg/m^2^ or mm	Water stored in/on the land surface vegetation at the date mentioned, but weighted such that it corresponds to an average height of water valid over the whole land surface
GEM-Hydro_calibrated_SWE.nc	SWE	kg/m^2^ or mm	Average Snow Water Equivalent over land surface at the date mentioned
GEM-Hydro_calibrated_WSL1.nc	WSL1	m^3^/m^3^	Soil moisture content for soil layer 1: for depth between 0 and 5 cm (volumetric fraction) at the date mentioned
GEM-Hydro_calibrated_WSL2.nc	WSL2	m^3^/m^3^	Soil moisture content for soil layer 2: for depth between 5 and 10 cm (volumetric fraction) at the date mentioned
GEM-Hydro_calibrated_WSL3.nc	WSL3	m^3^/m^3^	Soil moisture content for soil layer 3: for depth between 10 and 20 cm (volumetric fraction) at the date mentioned
GEM-Hydro_calibrated_WSL4.nc	WSL4	m^3^/m^3^	Soil moisture content for soil layer 4: for depth between 20 and 40 cm (volumetric fraction) at the date mentioned
GEM-Hydro_calibrated_WSL5.nc	WSL5	m^3^/m^3^	Soil moisture content for soil layer 5: for depth between 40 and 100 cm (volumetric fraction) at the date mentioned
GEM-Hydro_calibrated_WSL6.nc	WSL6	m^3^/m^3^	Soil moisture content for soil layer 6: for depth between 100 and 200 cm (volumetric fraction) at the date mentioned
GEM-Hydro_calibrated_WT.nc	WT	[—]	Fraction of grid cell occupied by land surface (constant over time).
GEM-Hydro_calibrated_streamflow.zip	Q	m^3^/s	Pairs of observed and simulated mean daily streamflow at flow gauge locations in txt format. Once unzipped, one file per gauge. The .txt filenames correspond to a US or Canadian gauge ID.

See Methods for more details about the computations performed with the actual GEM-Hydro outputs to produce the variables mentioned in this table. All dates in these files are in UTC format, except for streamflow for which daily averages correspond to the time interval between midnight and midnight of the day after in local time (always EDT or UTC – 4 h).

The equation to compute the SVS water storage for any given date is given below:

7 $$S={WVEG}+{SWE}+\sum _{i=1,6}{({WSL}}_{i}\ast {H}_{i})$$

where:

*S*: Total water stored in the SVS land-surface scheme for a given date, in mm or kg/m^2^.

${WVEG},{SWE},{{WSL}}_{x}$: The three different SVS variables related to water storage (see Table 5), in mm or kg/m^2^.

${H}_{i}$: Thickness of the ${i}^{{th}}$ SVS soil layer, in mm (see Table 5).

For the gridded surface variables (i.e. all except streamflow), the grid has a 0.09-degree resolution (~10 km) and a size of 191 × 143 grid cells, but the values are not provided outside of the aforementioned watersheds (see Methods for processing the GEM-Hydro outputs). These gridded variables are moreover only valid over the land fraction of a grid cell and not over other surface types like water, because they correspond to outputs of the GEM-Hydro’s LSS, named SVS (see Background and Summary). All the gridded surface variables of this dataset have therefore a value of 0.0 over pixels that are covered with 100% water in the domain (except soil moisture which has a value of 1.0for aesthetic purposes). Note that the subgrid-scale lakes were however filtered out and replaced with land areas in the GEM-Hydro setups used here (see Changes to the surface component in Methods), meaning that most of the grid cells of the region are assumed to be occupied at 100% by land, except around and in grid cells that are occupied by 100% water. More information about the reason for (and the impact of) neglecting the subgrid-scale lakes in the GEM-Hydro setups used here can be found in Changes to the surface component (Methods). Despite of this, the surface variables shared in this dataset can still be used to force any routing model, as explained in Usage Notes. The land cover fraction inside each grid cell does not evolve with time in this version of GEM-Hydro and is provided in this dataset as the “WT” variable (see Table 5).

The SM values shared in this dataset (see Table 5) correspond to liquid water only, because the version of SVS used during this study does not include the representation of soil freeze/thaw processes, and therefore does not simulate the conversion from liquid to frozen soil water during cold seasons. This is because when activating the soil freeze-thaw processes with SVS, spring freshets are generally strongly overestimated, partly because the model does not allow for infiltration into frozen ground, which could occur in reality because of macropores (Mohammed *et al*.,^46^). Work is under way to improve the representation of the soil freeze-thaw processes with SVS and their impact on resulting streamflow simulations.

Table 5 summarizes the different files shared in this dataset, as well as their content. Note that each file of this dataset contains a unique hydrologic variable over the region of interest and over the full period of interest, except for streamflow where the file shared is a compressed file. Once decompressed, there will be one text file for each of the 212 flow gauges considered in the domain, containing the daily observed and simulated streamflow over the full period.

## Technical Validation

### Streamflow performances

To perform the evaluation of GEM-Hydro streamflow simulations over the Great Lakes and Ottawa river basins, the mean daily GEM-Hydro simulated streamflows were computed at the location of the 212 streamflow gauges used in the GRIP-GL project (Mai *et al*.^15^), for which mean observed daily streamflow is available over the period from January 1^st^, 2001, to December 31^st^, 2017. Despite the GEM-Hydro simulations performed here also cover the year 2018, it was not considered in this evaluation because the observations were gathered from the data of the GRIP-GL project, which does not include the year 2018. Please refer to the sub-section “Methods for processing the GEM-Hydro outputs” for a justification of using a daily time-step to perform the streamflow evaluation.

**Fig. 5:**
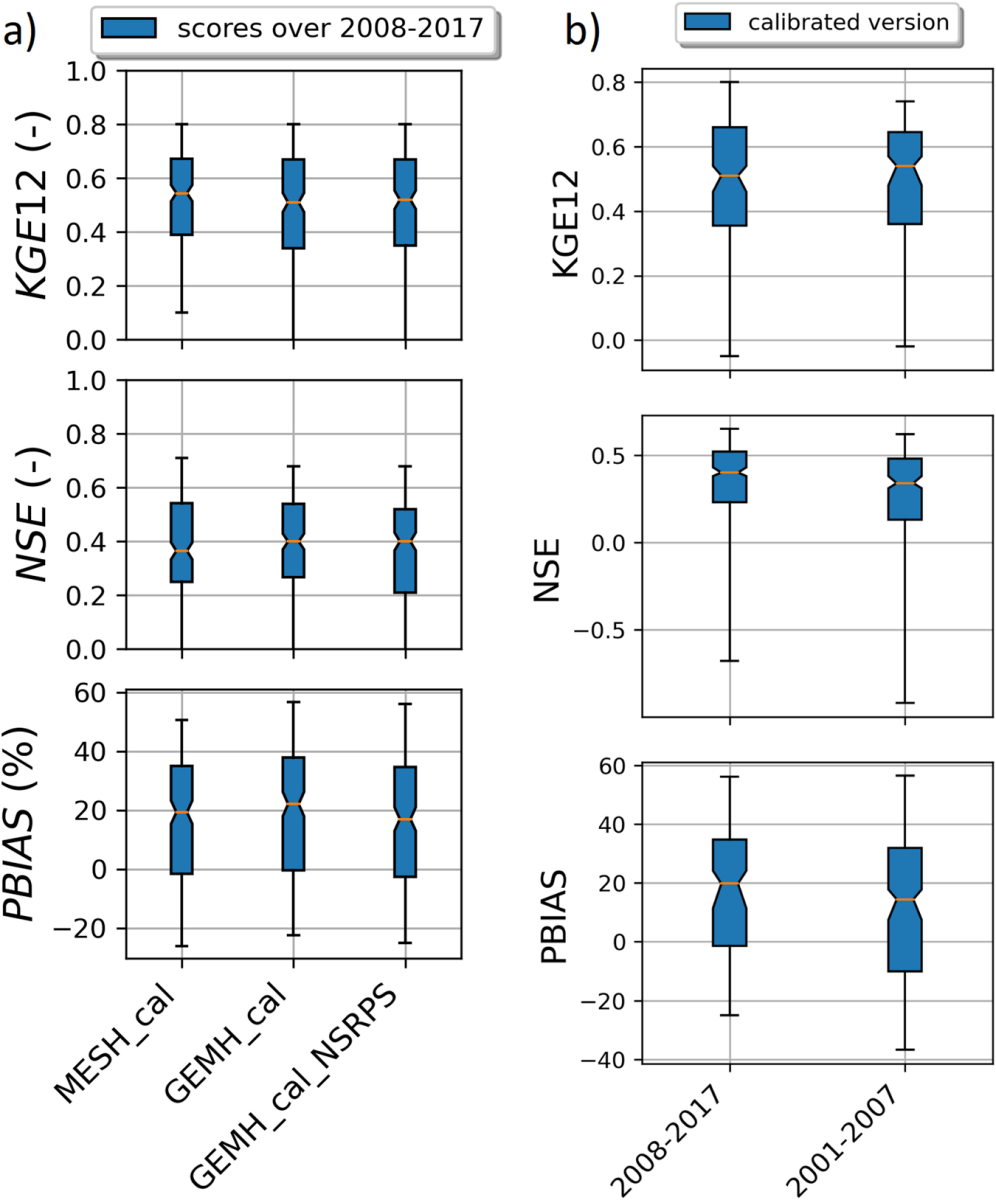
Boxplots of streamflow performances across the 212 flow gauges considered here, for the different calibrated models. (**a**) performances over the calibration period (1^st^ Jan. 2008- 31 Dec. 2017) for the three different calibrated model runs mentioned in this document. MESH_cal corresponds to the calibrated version of MESH-SVS-Raven using the GRIP-GL setup configuration, GEMH_cal corresponds to the calibrated version of GEM-Hydro using the GRIP-GL setup configuration, and GEMH_cal_NSRPS corresponds to the calibrated version of GEM-Hydro using the NSRPS setup configuration (see Methods and text for more details). (**b**) performances for the calibrated version of GEM-Hydro using the NSRPS configuration over the calibration (20080101-20171231) and validation (20010101-20071231) periods. The target value for KGE12 (revised KGE) and NSE criteria is 1.0, while the target value for PBIAS (relative percent bias) is 0.0. On Figs. 5 and 6, the lower and upper limits of the boxes correspond respectively to quartiles 25 and 75% of the 212 gauges’ performances, the median score value is shown with a horizontal line in the thinner part of the box, and the lower and upper whiskers correspond respectively to percentiles 5 and 95% of the gauges’ performances, while the outliers are not shown.

**Fig. 6:**
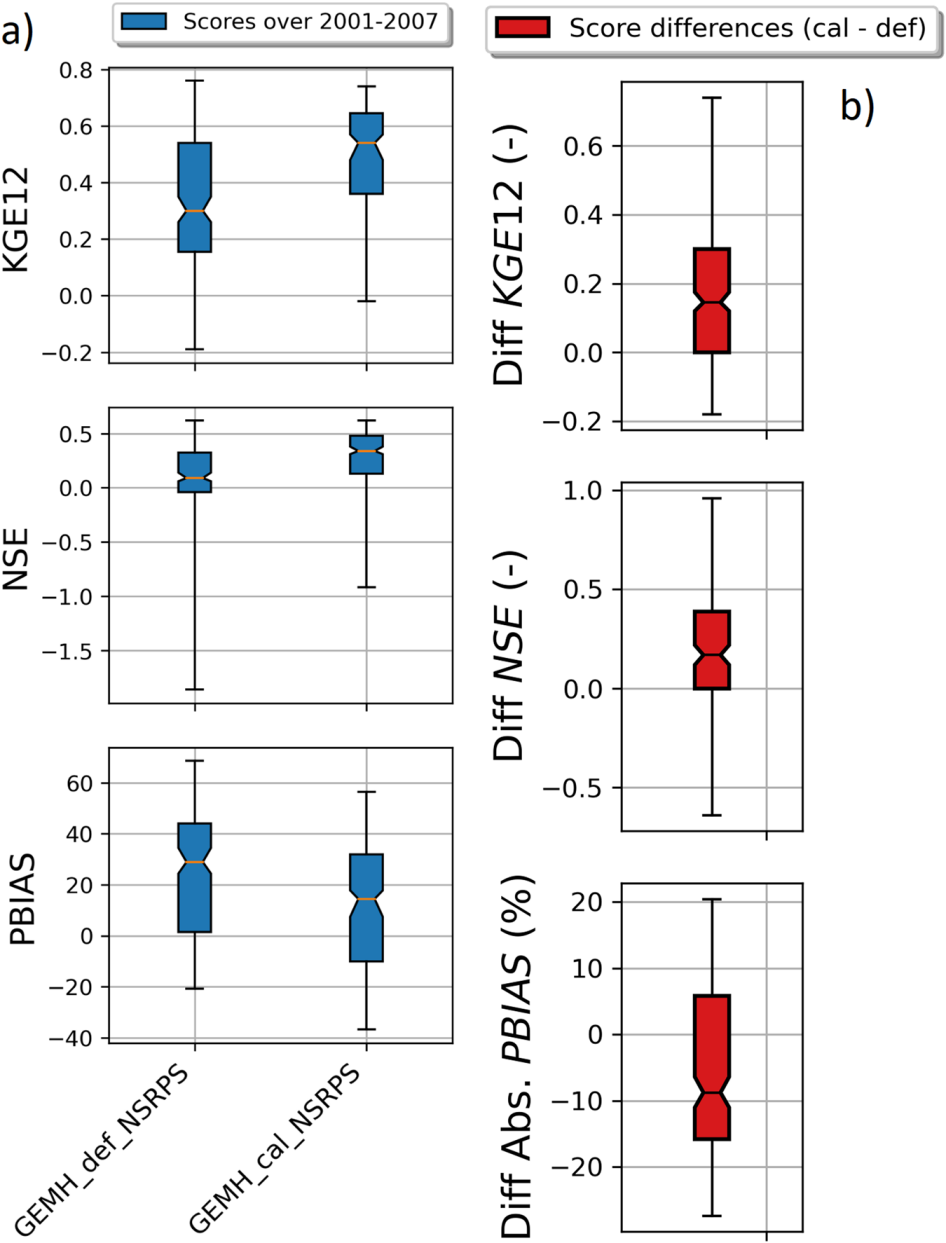
boxplots of streamflow performances across the 212 flow gauges considered here, for the default and calibrated versions of GEM-Hydro over the validation period (20010101-20071231) and using the NSRPS configuration. (**a**) Performances of each version separately. GEMH_def_NSRPS corresponds to the default GEM-Hydro version. GEMH_cal_NSRPS corresponds to the calibrated version of GEM-Hydro. (**b**) Differences in streamflow performances between the two versions of GEM-Hydro. For the KGE and NSE criteria, the difference between the calibrated and default versions of GEM-Hydro was computed at each gauge location, and values above 0.0 indicate an improvement of the calibrated upon the default version of the model. For the PBIAS criteria, the difference between the calibrated and default version of the model was computed using the absolute PBIAS value of each version and at each gauge location, and negative values indicate an improvement of the calibrated upon the default version of the model. See legend of Fig. 5 for more details on the scores and the boxplots shown here.

For a description of the flow gauges’ main attributes (gauge ID, river name, drainage area, mean elevation and mean annual runoff, etc.), please refer to the supplementary material of GRIP-GL (Mai *et al*.^15^). The subbasins corresponding to these 212 flow gauges are shown on Fig. 2. Figure 5 and 6 show boxplots of streamflow performances across these 212 flow gauges, either over the calibration (2008/01/01-2017/12/31) or validation period (2001/01/01-2007/12/31), for MESH-SVS-Raven and different versions of GEM-Hydro, and for three different scores. The scores include the revised KGE criteria (see Kling *et al*.^39^), the Nash-Sutcliffe criteria (NSE, see Nash and Sutcliffe, 1970^47^) and a relative percent bias criterion (see Eq. 8 below).

8 $${PBIAS}=\frac{\mathop{\sum }\limits_{i=1}^{N}({O}_{i}-{S}_{i})}{\mathop{\sum }\limits_{i=1}^{N}{O}_{i}}\ast 100$$

where:

${PBIAS}$: Relative Bias in percent

${O}_{i}$: Observed streamflow for day *i*

${S}_{i}$: Simulated streamflow for day *i*

Therefore, when the *PBIAS* value is positive (negative), it denotes an underestimation (overestimation) of the observed flows by the simulations.

First, it can be seen on Fig. 5a that when the SVS and some routing parameters are calibrated with MESH-SVS-Raven and then transferred into GEM-Hydro, the streamflow performances remain relatively similar, highlighting the relevance of the approach used here to calibrate SVS and some routing parameters using another system than GEM-Hydro. The same is true when modifications are made to the GEM-Hydro setup to use a configuration that is more representative of the one used in the NSRPS system. This is encouraging because it supports the idea that the calibrated parameters obtained here with MESH-SVS-Raven were not overfitted to the specific setup configuration used with MESH-SVS-Raven and can be transferred to a GEM-Hydro version using a different setup configuration. The absence of over-calibration or overfitting in the calibrated parameter values obtained here is moreover supported by the evaluation of the auxiliary hydrologic and near-surface variables presented further down.

It can be seen on Fig. 5b that the calibrated version of GEM-Hydro displays a strong temporal robustness, because the streamflow performances are generally similar between the calibration and validation periods. Finally, it can be seen on Fig. 6 that the calibrated version of GEM-Hydro generally displays better streamflow performances than the default version of the model (without calibration), with a median KGE improvement close to 0.2 (see Fig. 6b).

**Fig. 7:**
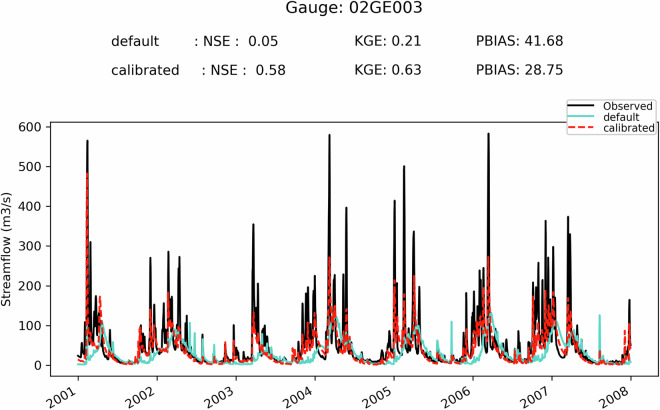
Hydrographs for gauge 02GE003, for the default and calibrated versions of GEM-Hydro, over the validation period. The flow observations are shown with the black line. The station 02GE003 is located on the northern shore of Lake Erie, in an agricultural area, and has a drainage area of 4498 km^2^.

**Fig. 8:**
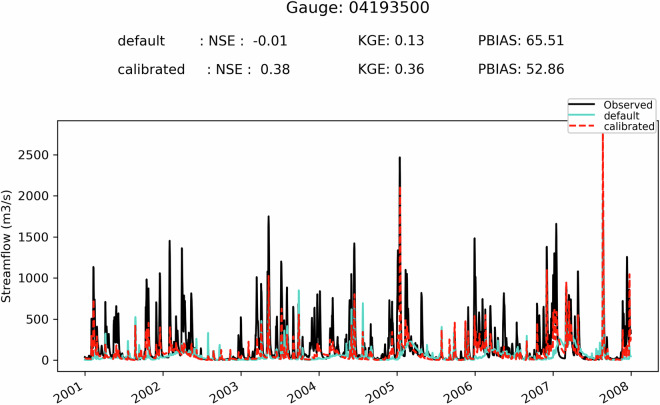
Hydrographs for gauge 04193500, and for the default and calibrated versions of GEM-Hydro, over the validation period. The flow observations are shown with the black line. This basin is in the southwestern part of the Lake Erie subdomain, in an agricultural area, and has a drainage area of 16395 km^2^.

**Fig. 9:**
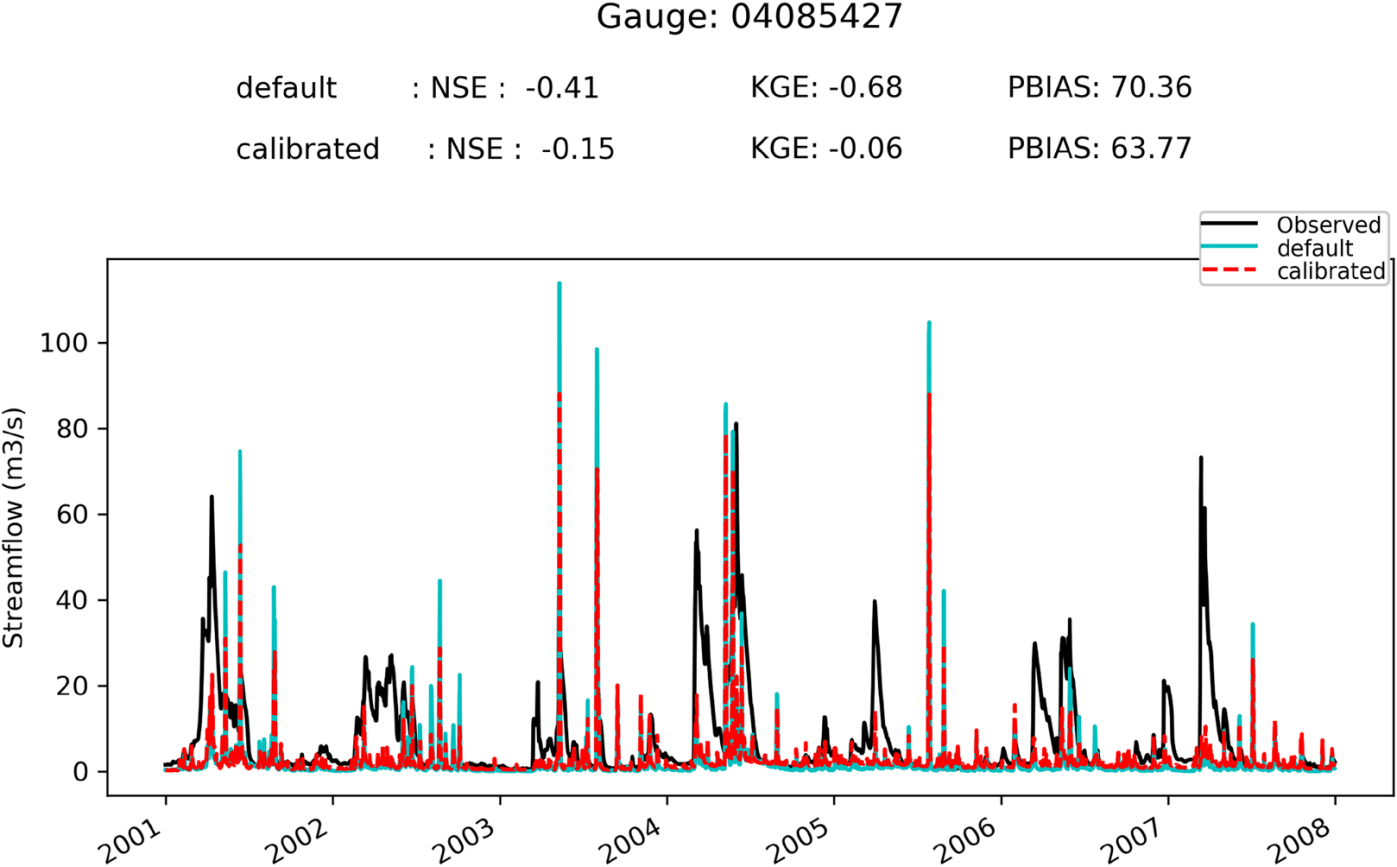
Hydrographs for gauge 04085427, and for the default and calibrated versions of GEM-Hydro, over the validation period. The flow observations are shown with the black line. This basin is located on the western side of Lake Michigan and has a drainage area of 1362 km^2^.

**Fig. 10:**
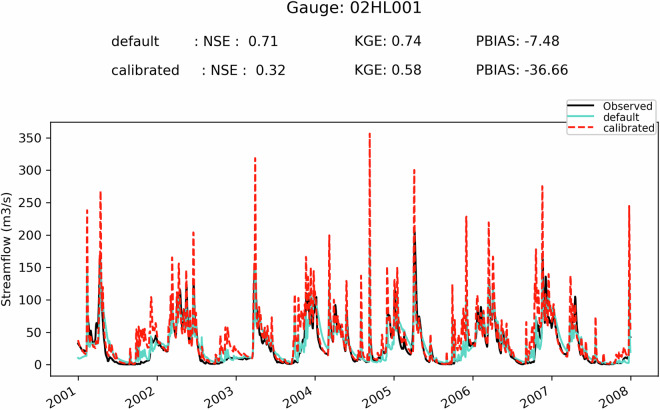
Hydrographs for gauge 02HL001, and for the default and calibrated versions of GEM-Hydro, over the validation period. The flow observations are shown with the black line. The station 02HL001 is located on the northern shore of Lake Ontario, in an agricultural area, and has a drainage area of 2673 km^2^.

**Fig. 11:**
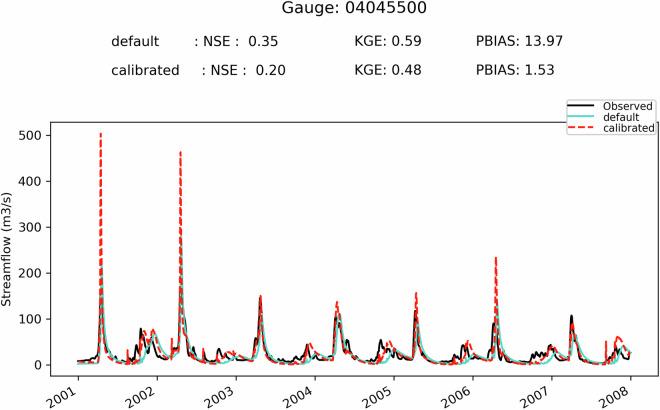
Hydrographs for gauge 04045500, and for the default and calibrated versions of GEM-Hydro, over the validation period. The flow observations are shown with the black line. This basin is in the southeastern part of Lake Superior domain and has a drainage area of 2046 km^2^.

It can also be seen on Fig. 6b that regarding the KGE and NSE criteria, an improvement occurred for 75% of the 212 flow gauges considered. However, it also means that for 25% of the stations, the calibrated version of GEM-Hydro degrades flow performances compared to the default version of the model. When looking at flow hydrographs, this was generally attributed to overestimated streamflow peaks and flow volumes, with the calibrated version. This is supposed to be caused by the calibrated parameters obtained here, which imply a significant increase in the horizontal and vertical hydraulic conductivities in agricultural areas, especially for the watersheds of Lake Erie and Lake Ontario. This is because GEM-Hydro is missing an explicit representation of the tile drains in these areas, and because of the strategy employed here to represent the effect of these tile drains in the model (see Methods). Indeed, with this strategy, tile drains are assumed to be present in 100% of the agricultural areas, which is probably not the case. Where tile drains are present, the calibration methodology employed here therefore probably leads to an improvement of the simulations compared to the default version of the model (but still to generally underestimated flow volumes, see for example Figs. 7–9). When tile drains may not be present, the calibrated parameters however probably lead to overestimated flows (see Figs. 10, 11). The final calibrated values for GRKMO_A and KASMO_A (see Fig. 3) therefore probably consist of a trade-off between agricultural areas that are strongly impacted by human influence, and those that are less influenced. This may be improved in the future for example by using a GRKMO_A calibration parameter that would only be tied to areas where tile drains are present, and a KASMO_A calibration parameter only tied to areas where significant ploughing practices occur, instead of being applied to all agricultural areas. This information would however need to be available on maps (over the US and Canada), which was not the case at the time of this study, to the extent of our knowledge. This is however dedicated to future work. For the time being, we consider that the calibrated version of GEM-Hydro, whose outputs are described here, generally represents an improvement upon its default counterpart, for most of the Great Lakes and Ottawa River watersheds.

**Fig. 12:**
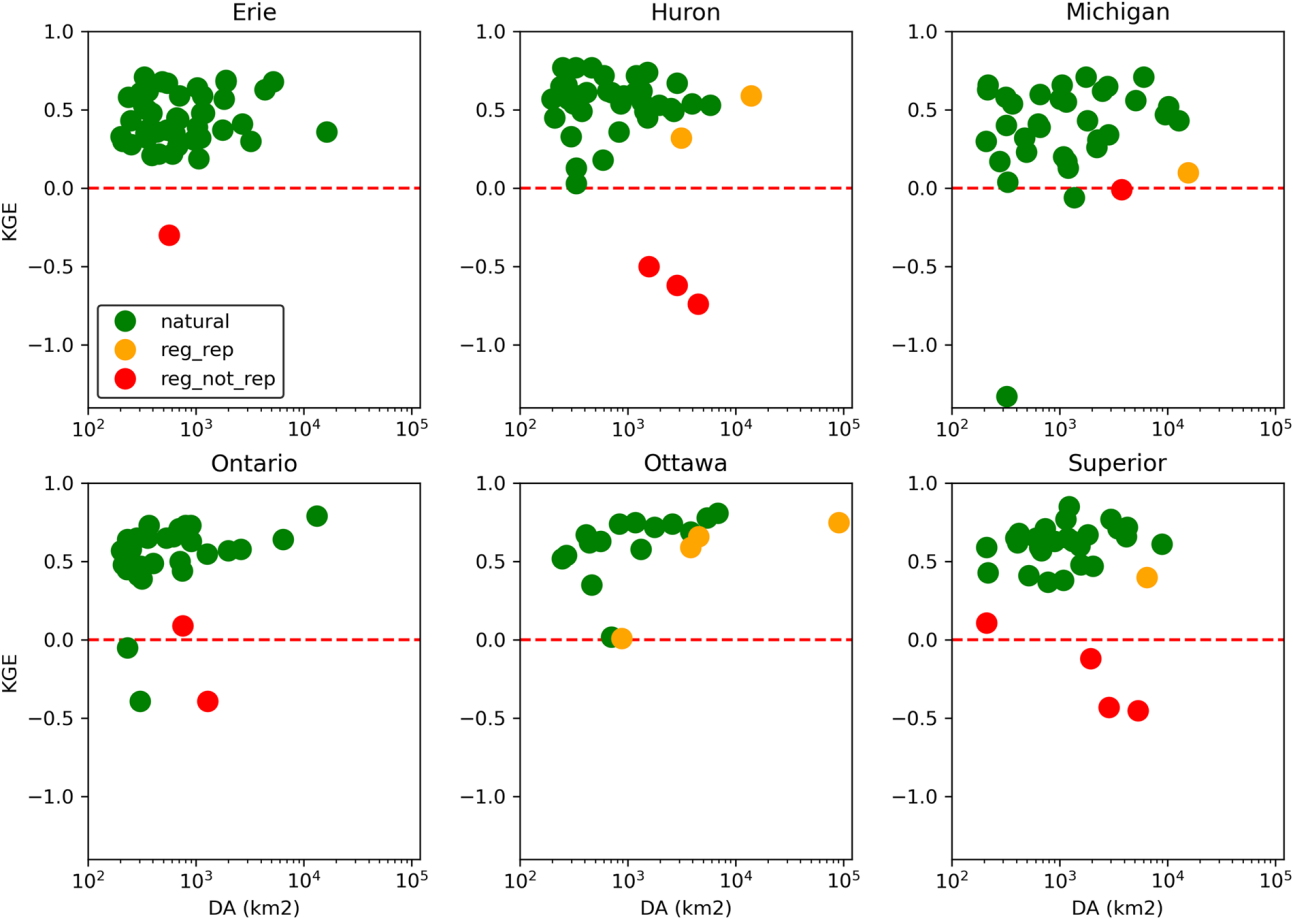
Overview of the streamflow performances (KGE) of the calibrated GEM-Hydro version (NSRPS configuration), over the validation period. Performances are shown for each subdomain separately, and as a function of basin size (DA, in km2 – logarithmic scale). Green dots (“natural”) represent natural basins. Orange dots (“reg_rep”) represent basins that are regulated and for which the regulation is explicitly represented in GEM-Hydro based on the DZTR reservoir model. Red dots (“reg_not_rep”) represent regulated basins for which the regulation is not explicitly represented in GEM-Hydro. Note that some of the green dots may correspond to red ones, since not all stations shown here were carefully checked for their regulation type, but only the ones with low performances inside each basin.

It can be seen from Fig. 12 that there is no clear relationship between streamflow performances of a given basin and its size, even if there seems to be some tendency for performances to display less variability and to increase slightly with the basin size, especially when excluding regulated catchments. Moreover, basins for which the regulation is explicitly represented in GEM-Hydro using the DZTR reservoir model do not show performances significantly different than those from other “natural” basins. Finally, there also doesn’t seem to be a subdomain for which streamflow performances are clearly better or worse than the general model performances depicted on Fig. 6a) (Median performances of about 0.5 in terms of KGE). There is however a clear signal that some of the basins with the worst performances correspond to regulated catchments for which the regulation is not explicitly represented in GEM-Hydro.

### GEM-Hydro auxiliary Hydrologic variables

In order to evaluate the performances of the calibrated version of GEM-Hydro beyond streamflow performances, the default and calibrated versions of GEM-Hydro (using the final NSRPS configuration) were compared with regard to the performances of auxiliary hydrologic variables, as was done in GRIP-GL (Mai *et al*.^15^), but also from the viewpoint of near-surface meteorological variables (see further down). This is important to evaluate a physically-based model with other variables than streamflow especially in the case where the model was calibrated to maximize only streamflow performances. This indeed allows to make sure that the calibration process does not result in degrading other physical processes in the model (Kirchner 2006^48^). It can indeed often happen with calibration that the final performances are good regarding streamflow but imply unrealistic physical processes. This is in opposition to the idea of “getting the right answer for the right reasons” (Kirchner 2006^48^). Getting the right answer for wrong reasons is linked with the notion of parameter equifinality (Beven and Binley 1992^49^): very different parameter sets, all leading to similar streamflow performances, can imply very different internal physical processes, some of which may be unrealistic to various degrees. This is especially important to assess the performances of the calibrated SVS version for other variables in the context where this LSS could ultimately be two-way coupled with an atmospheric model, as mentioned in Methods.

The goal here is therefore to make sure that the performances of the calibrated version of GEM-Hydro remain similar or are better than the default version of the model, when looking at other outputs of the model. Here we focus on three hydrologic variables other than streamflow: the model total ET over land (which in GEM-Hydro is the sum of bare ground evaporation and vegetation ET, in mm), the SSM (mean soil moisture between 0 and 10 cm depth, in m^3^/m^3^), and the mean SWE on the ground (in mm). To evaluate these variables, their mean daily value was computed from the hourly GEM-hydro outputs (or the total value in the case of AET) and compared to a reference dataset for each of them. The total AET over land is a direct output of GEM-Hydro that is shared in this dataset, the mean SVS SWE was computed as specified in Methods for processing the GEM-Hydro outputs, and the SSM between 0 and 10 cm was simply computed by taking the average of soil moisture for the first two SVS soil layers.

The reference datasets are mentioned in Table 6. They are the same as those used during GRIP-GL (Mai *et al*.^15^). Note that these datasets do not consist of purely observed data such that they cannot be considered as the “truth” for these variables. The idea here is, however, not to perform a comprehensive evaluation of the calibrated version of GEM-Hydro, but rather to compare it to the default version of the model to make sure that the performances remain at least similar between the two. Note that regarding ERA-5 Land that is used as the reference for SWE, it was preferred upon *in-situ* measurements of SWE because these *in-situ* measurements are not gridded (they are not available for all pixels of the region) and are not available daily either. However, an evaluation of the calibrated GEM-Hydro version and ERA-5 Land against *in-situ* SWE measurements is still performed here.

**Table 6:** List of the reference datasets used to evaluate the GEM-Hydro auxiliary hydrologic variables.

Variable	Reference dataset
Evapo-transpiration, in mm	Global Land Evaporation Amsterdam Model (GLEAM) v3.5b (see https://www.gleam.eu/ and Martens *et al*.^52^)
Soil moisture (0–10 cm), in m^3^/m^3^	GLEAM v3.5b (see https://www.gleam.eu/ and Martens *et al*.^52^)
Snow water equivalent (mm)	ERA-5 Land (10.24381/cds.e2161bac) See also Muñoz-Sabater^13^

The evaluation against these reference datasets was performed by computing the KGE criteria for each grid cell of the full domain, using the time-series of mean daily (or total daily for AET) values of these variables. Note that in this case, the KGE values correspond to the original (not revised) formulation of the KGE, as proposed by Gupta *et al*.^50^. For AET and SSM, the period from 2003 to 2017 was used because this is the period for which the GLEAM v3.5 data were available (Table 6), but the period from 2001 to 2017 was used for the comparison against ERA-5 Land regarding SWE. Finally, the GEM-Hydro outputs were re-gridded to the resolution of the reference datasets, which is of 0.25° for the GLEAM dataset (AET and SSM), and of 0.1° for ERA-5 Land (SWE). See Mai *et al*.^15^ for more details on the auxiliary variables’ evaluation.

**Fig. 13:**
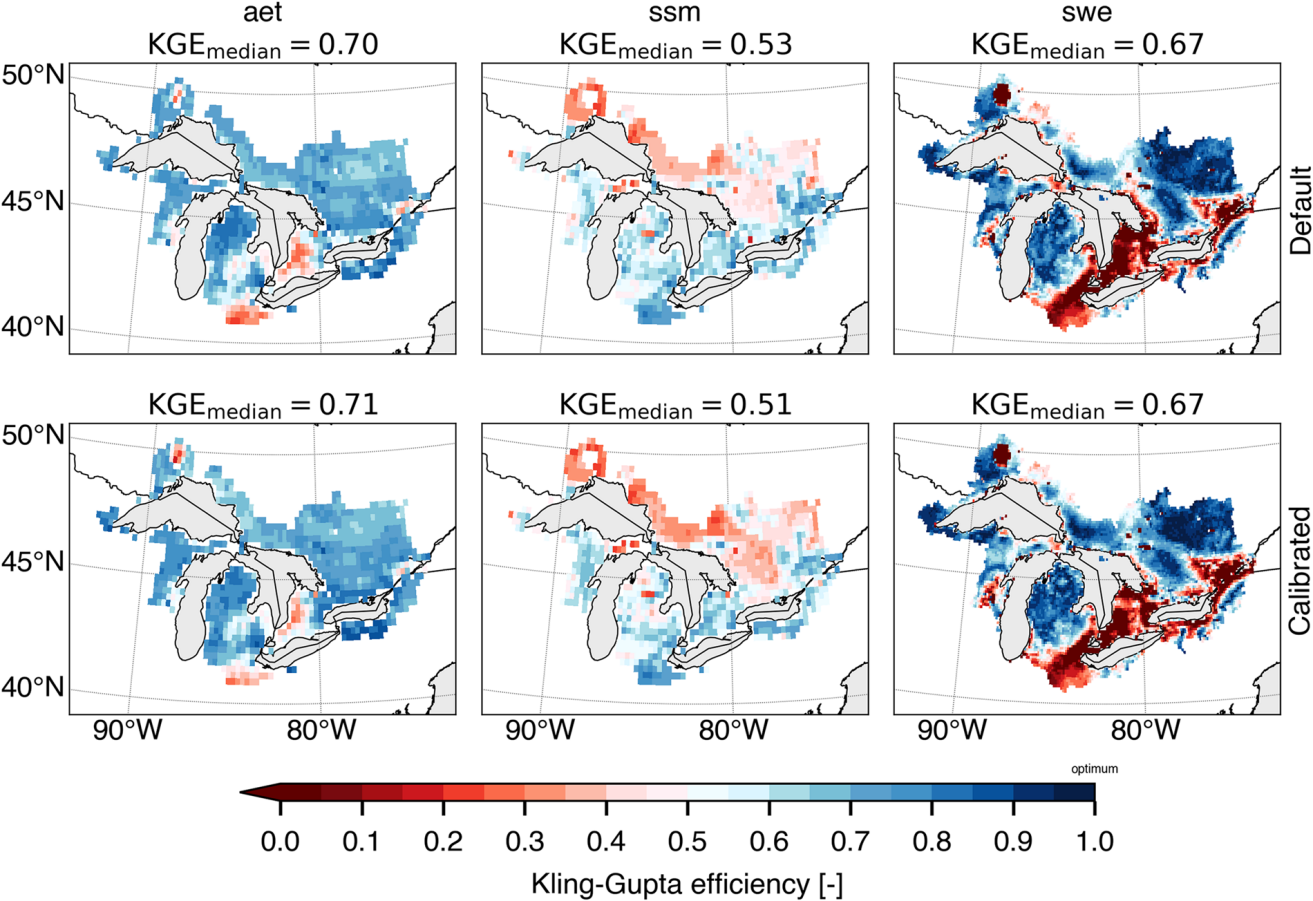
Comparison of the two versions of GEM-Hydro against reference datasets for the auxiliary hydrologic variables. The top row shows the comparison for the default GEM-Hydro version, while the comparison of the calibrated version is shown on the bottom row). The three auxiliary hydrologic variables considered here consist of evapo-transpiration (aet, left column), superficial soil moisture (ssm, middle column), and snow water equivalent (swe, right column).

It can be noticed on Fig. 13 that the default and calibrated versions of GEM-Hydro generally display similar performances when compared to the reference datasets used here. Note that to achieve these results, a total of 6 successive calibration trials were performed (see Methods), during which several modifications to the calibration methodology were applied to address degradations that were sometimes noticed with the calibrated version for some auxiliary variables and in some regions. As previously mentioned, this is because of the equifinality issue that especially arises when only calibrating a physical model to streamflow. Note that performing multi-objective calibration to include for example these three auxiliary variables in the OF during calibration would however not be a trivial task that could also call for several iterations of the procedure (see Mai 2023^29^), and which could also lead to suboptimal streamflow performances (Mei *et al*.^31^). Maximizing streamflow performances was however the main objective of the calibration work performed here, to ultimately improve the NSRPS real-time streamflow forecasts performed at ECCC. This is why it was preferred to only target flow performances during calibration and evaluate the model based on other variables afterwards.

It can be noticed on Fig. 13 that for each of the three GEM-Hydro auxiliary variables, and whatever the version of the model considered (i.e. default or calibrated), there are some areas that display a stronger discrepancy than others against the reference data used here. For AET the areas with stronger discrepancy consist mostly of the northern shore and the southern west portion of Lake Erie, which are mainly covered with agricultural areas. Both GEM-Hydro versions tend to simulate higher AET than the reference dataset in these areas, which was diagnosed by looking at the bias component of the KGE (not shown here). It was noticed that a better match between the calibrated GEM-Hydro version and the reference dataset was obtained in these areas for AET when using much higher values (close to 100.0) for the KASMO_A parameter (used to represent the effect of ploughing in agricultural areas, see Table 1). But in this case, there was however a stronger mismatch in these areas for SSM. It was therefore preferred to limit the variation of the KASMO_A parameter to a maximum value of 5.0 (Table 1) to remain closer to the default version of the model. Agricultural areas are the subject of many complex anthropogenic effects such as ploughing, the presence of Tile drains, and irrigation, none of which is currently explicitly represented in GEM-Hydro. It is therefore challenging to make sure that better results are obtained in these areas for good reasons regarding auxiliary variables, when using a calibrated version of GEM-Hydro.

Regarding SSM, the areas with the strongest discrepancies between GEM-Hydro and the reference dataset consist of the northern part of the Great Lakes region. While it is not exactly known why, it must be emphasized that these areas correspond to regions mostly covered with high vegetation, where the remotely sensed (satellite) SM data (that GLEAM assimilates) are known to be less accurate (Tong *et al*.^51^). Therefore, it is not sure that GEM-Hydro performs worst in this northern region with regard to SSM. It is also emphasized that the actual KGE performances are displayed here for SSM, while in GRIP-GL only the correlation component of the KGE was shown for this variable, given that many models were not actually simulating SSM. It is however much easier to achieve satisfactory SSM simulations when only looking at correlation, rather than when looking at the actual KGE value as done here. Finally, regarding SWE, there are several areas showing a strong discrepancy between GEM-Hydro and ERA-5 Land.

**Fig. 14:**
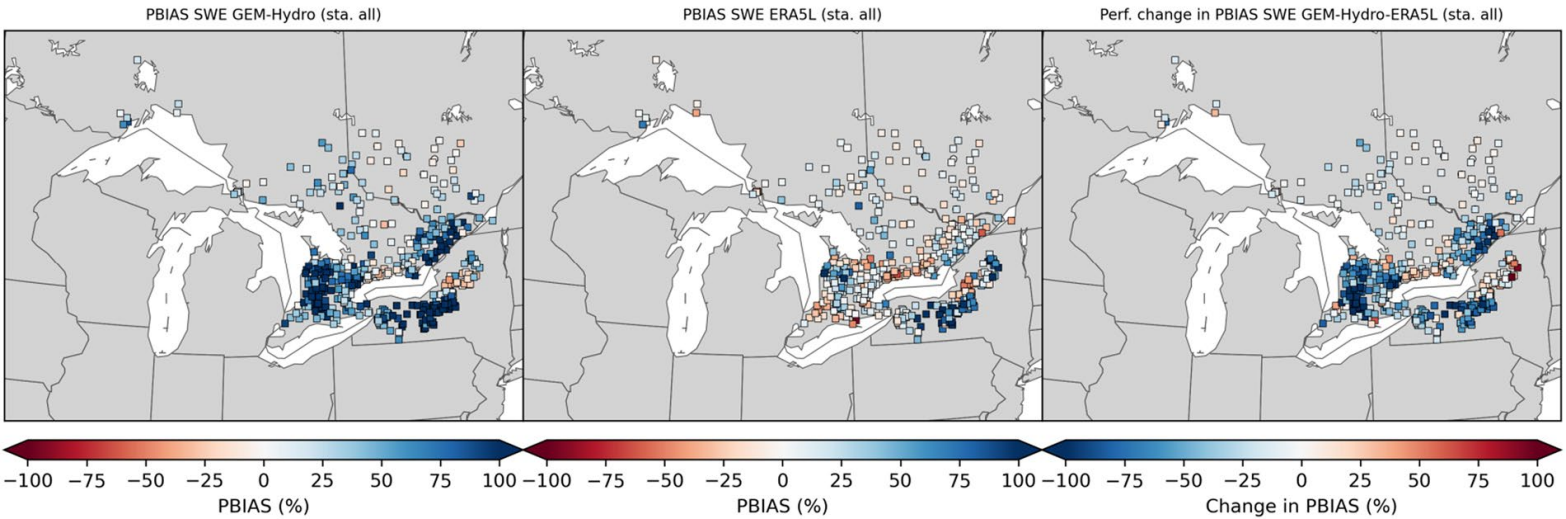
Comparison between the bias of SWE simulations from GEM-Hydro and from ERA-5 Land, over the watersheds considered in this study and over the period from October 1st, 2001, to October 1st, 2018 (17 winters). The observations mainly consist of in-situ manual snow surveys included in the CanSWE database (Vionnet *et al*.^53^) and taken from the northeastern US databases (Mortimer *et al*.^54^). Only stations with at least 20 observations (~one average per winter) available were considered. For the left (GEM-Hydro bias) and middle (ERA-5 Land bias) panels, the bias shown consists of the relative bias expressed in % (see Eq. 8) but using the difference between the simulated and observed values, in opposition to Eq. 8 (see text for more details). Blue (red) colors imply an over- (under-) estimation of the observed values. The differences between the absolute PBIAS values of ERA-5 Land and GEM-Hydro are shown on the right panel, such that red (blue) colors indicate that GEM-Hydro SWE simulations have less (more) bias than those of ERA-5 Land.

For the areas exhibiting strong differences between ERA-5 Land and GEM-Hydro in terms of SWE simulations (see Fig. 13), it can be seen on Fig. 14 that ERA-5 Land is generally better than GEM-Hydro. The strong positive *PBIAS* values observed for GEM-Hydro SWE simulations in some areas of Fig. 14 correspond well to the areas for which strong differences were noticed between GEM-Hydro and ERA-5 Land, in terms of SWE (see Fig. 13). This SWE overestimation by GEM-Hydro (see legend of Fig. 14) in these areas was less pronounced (but still present) when using the traditional 0°-threshold method to separate liquid and solid precipitation in GEM-Hydro (not shown), instead of the Harder and Pomeroy (2013^41^) method used here for the precipitation PPM (see Methods). The evaluation of the 0°-threshold method has however shown that it creates a strong understimation of snowfall occurrence for many regions of Canada (see for example Vionnet *et al*.^42^). On the other hand, evaluation of the precipitation PPM from Harder and Pomeroy (2013^41^) over the Great Lakes has shown that it can lead to an overestimation of snowfall occurrence (not shown here). It is therefore supposed that the positive bias noticed here with GEM-Hydro regarding SWE for some areas is a combined result from a positive bias in CaSR v2.1 winter precipitation forcings (Gasset *et al*.^14^) and a positive bias in snowfall occurrence from the PPM of Harder and Pomeroy (2013^41^). Additional investigations are required in the context of the preparation of the CaSR v3.0 and are beyond the scope of this document. There are areas for which the GEM-Hydro simulations performed here are however very competitive with (or very close to) ERA-5 Land regarding SWE simulations (see Figs. 13, 14).

### GEM-Hydro near-surface meteorological variables

As explained in Methods, it is also important to make sure that the calibrated version of GEM-Hydro does not degrade the near-surface variables simulated by the model, as compared to the default version. This is related to the fact that ultimately, the calibrated version of the surface component of GEM-Hydro could be two-way coupled with ECCC atmospheric models (see Model evaluation regarding streamflow and auxiliary variables in Methods). The three surface variables considered here consist of 2-m air temperature (TT, in °C.), 2-m dew point (TD, in °C.), and 10-m wind speed (UV, in m.s^−1^). It is possible to evaluate these GEM-Hydro variables because they are simulated by the model and do not consist of forcing variables. Indeed, GEM-Hydro is driven with atmospheric forcings (like air temperature and humidity and wind) corresponding to the lowest prognostic level of an atmospheric model, which in the case of the CaSR v2.1, corresponds approximately to 40 m. To perform the evaluation of the default and calibrated versions of GEM-Hydro regarding these variables, the ECCC internal verification tool “EMET” was used. The evaluation was performed by considering *in-situ* observations from the METAR, SYNOP, and SWOB observation networks over the full domain considered here, using the hourly GEM-Hydro outputs. Each one of the two GEM-Hydro versions was evaluated by computing the mean bias and the standard deviation of the error of the simulations, as compared to the observed values of a given variable and over the period from 2013 to 2017 included. The average of a given score was then computed over the area of interest. Then, the performances of the two versions were compared and are shown in Table 7, which shows the relative differences between the performances of the two versions of GEM-Hydro (i.e., default and calibrated).

**Table 7:** Comparison between the default and calibrated versions of GEM-Hydro regarding surface variables.

	Period/variable	FULL	WINTER	SPRING	SUMMER	FALL
		20130101/20171231	0101–0331	0401–0630	0701–0930	1001–1231
REL. ΔBIAS	TD	1.45%	1.06%	2.50%	1.30%	1.02%
	TT	1.00%	−0.28%	1.40%	0.37%	0.15%
	UV	−0.27%	0.12%	−0.49%	−0.41%	−0.17%
REL. ΔSTDEV	TD	0.08%	−0.03%	0.13%	0.04%	0.01%
	TT	−0.05%	−0.03%	−0.06%	0.02%	0.01%
	UV	−0.02%	−0.01%	−0.04%	−0.04%	−0.02%

TD: 2-m dew point temperature. TT: 2-m air temperature. UV: 10-m wind speed. Relative differences of the bias and the standard deviation of the error between the two experiments are shown. Positive values denote an improvement of the calibrated version upon the default one, while negative values denote a degradation. Underlined values correspond to the most significant differences between the two versions. Note that the relative differences are shown here for different periods. For the values split by season, note that for each season, the average of the values for the years 2016 and 2017 is shown. See text for more details on the computation of the relative differences shown here.

To compute the relative bias differences, Eq. 9 below was used:

9 $${REL}.\varDelta {BIAS}=\frac{\left|S2\right|-\left|S1\right|}{\left|S2\right|}\ast 100$$

where:

${REL}.\varDelta {BIAS}$: Relative BIAS difference (in %)

$S2$: Mean Bias of the default version of GEM-Hydro

$S1$: Mean Bias of the calibrated version of GEM-Hydro

The equation of the standard deviation of the error is given below.

10 $${STD}=\sqrt{\frac{1}{N}\ast \mathop{\sum }\limits_{i=1}^{N}{({X}_{i}-\bar{X})}^{2}}$$

where:

${STD}$: Standard deviation of the error

$N$: Total number of observation-simulation pairs.

${X}_{i}={P}_{i}-{O}_{i}$, where ${P}_{i}$ is the simulated (or forecasted) value for time-step *i*, and ${O}_{i}$ is the observed value for time-step *i*.

As such, the standard deviation of the error can be seen as a measure of the variations of the model errors from which the mean bias would have been removed.

To compute the relative difference of the standard deviation of the error, Eq. 11 below was used.

11 $${REL}.\varDelta {STDEV}=\frac{{S}_{2}-{S}_{1}}{{S}_{2}}\ast 100$$

where:

${REL}.\varDelta {STDEV}$: Relative difference of the standard deviation of the error (in %).

$S2$: Standard deviation of the error of the default GEM-Hydro version

$S1$: Standard deviation of the error of the calibrated GEM-Hydro version

It can be seen from Table 7 that the differences regarding near-surface variables are generally small between the two versions of GEM-Hydro and can be considered neutral in terms of the standard deviation of the error. A generally small improvement of the calibrated upon the default version can be noticed for TT and TD Bias, while small degradations are noticed for the wind speed. Note that regarding TT, the conclusions depend on the season considered. Regarding wind speed, it is not exactly known why a small degradation could occur with the calibrated version given that the multiplying coefficient related to surface roughness was not employed in this final calibration framework (see Methods). The UV bias differences are however generally very small between the two versions (of the order of 0.015 m.s^−1^, not shown here), which can be considered negligible. The same is true regarding differences related to TT and TD. The best improvement that was noticed for the spring season and for TD Bias (Table 7) for example involves maximum differences between the two GEM-Hydro versions of the order of 0.05 °C. (not shown here), which can also be considered negligible. Note that the model however tends to overestimate TD by 1 to 2 °C during the day (local time corresponds to UTC -4 hours during the spring period), over this region and for the spring season of 2017. When looking at the TD bias evolution as a function of the hour of the day but when considering the full period from January 1^st^, 2013, to December 31^st^, 2017, this TD overestimation however reaches 1 °C. (not shown). Regarding 2-m air temperature (TT), GEM-Hydro simulations however tend to underestimate this variable by about 0.5 °C. during the night (not shown).

## Usage Notes

It is emphasized here that the gridded surface variables shared in this dataset correspond to outputs of the SVS model, which is the land surface scheme of the GEM-Hydro model. Therefore, these surface variables are valid over land only, and not over other types of continental surfaces. Most grid-cells of the region of interest were however assumed to be covered only with land surfaces. See Changes to the surface component (in Methods) and Data records for more information about this. The surface fluxes shared in this dataset (including surface runoff, soil lateral flow, and soil base drainage) can therefore still be used as inputs to any routing model implemented over a basin that is included in the region of interest, provided that this basin does not include grid-cells that are filled at 100% with water surfaces: otherwise, the routing model will miss the fluxes coming from these 100% water pixels. This can be ensured based on the “WT” variable shared in this dataset, which represents the percentage of the land surface in each grid-cell (see Data Records), for the GEM-Hydro simulations performed here.

In order to drive a routing model with the surface fluxes shared in this dataset, the sum of surface runoff and lateral flow (i.e. the “TRAF” and “ALAT” variables of this dataset) should be directly given as inputs to the surface network of the routing model (i.e. lakes and rivers), while the SVS soil base drainage (the “O1” variable) should be provided first to a baseflow model (i.e., a model representing the aquifer), that is sometimes already included in the routing model. Indeed, the “O1” variable represents the aquifer recharge. The aquifer model will then simulate the baseflow that returns to the surface network of lakes and rivers, in the routing model. Note that the units of these surface fluxes correspond to kg/m^2^/h or mm/h (assuming a density of 1000 kg/m^3^, i.e. unsalted water). Therefore, when provided to a routing model, these fluxes should then be multiplied by a surface area (like the area of a subbasin or of the routing model grid-cell) to convert them into a volume of water per units of time. This is generally done in the routing model itself.

Finally, it is reminded here that the SM variables of this dataset (the WSL1-6 variables) only represent the liquid soil moisture content of a given soil layer. The version of SVS used in this work did however not represent soil freeze-thaw processes (more information in Data Records), such that the WSL1-6 variables still represent the total water stored in the different soil layers, in the GEM-Hydro simulations performed during this work.

## Data Availability

The SVS land-surface scheme and the WATROUTE routing scheme are both available in the MESH official repository available at this address: https://github.com/MESH-Model/MESH-Releases (accessed on February 1^st^, 2024). The Raven routing model is open-source and can be accessed here: the Raven Hydrological Framework - Home Page (uwaterloo.ca) (accessed on November 30, 2023). The DDS calibration algorithm is available in the Ostrich calibration toolkit and can be accessed here: OSTRICH Optimization Software Toolkit (uwaterloo.ca) (accessed on November 30, 2023). The MESH-SVS-Raven setups used in this study to calibrate the SVS and routing parameters may be shared upon reasonable request. GEM-Surf (the surface component of GEM-Hydro) is open-source and is available on Github: https://github.com/ECCC-ASTD-MRD/sps.git GEM-Surf can be run outside of ECCC informatic infrastructure. However, it still needs to rely on forcing and geophysical fields in the “standard file” format (a binary file format only used internally at ECCC), and produces output files in this format as well. Some tools needed to manipulate and read files of this format are also available on github: https://github.com/ECCC-ASTD-MRD. The WATROUTE version included in MESH can moreover not be run in a standalone mode, but only together with the SVS land-surface scheme included in MESH. The WATROUTE version used internally at ECCC cannot yet be run outside of ECCC infrastructure. It is therefore not yet possible to exactly replicate the GEM-Hydro simulations (I.e., by running GEM-Surf + WATROUTE) described here, outside of ECCC informatic infrastructure.
